# Soluble ICAM‐1 a Pivotal Communicator between Tumors and Macrophages, Promotes Mesenchymal Shift of Glioblastoma

**DOI:** 10.1002/advs.202102768

**Published:** 2021-11-23

**Authors:** Ki‐Chun Yoo, Jae‐Hyeok Kang, Mi‐Young Choi, Yongjoon Suh, Yi Zhao, Min‐Jung Kim, Jong Hee Chang, Jin‐Kyoung Shim, Seon‐Jin Yoon, Seok‐Gu Kang, Su‐Jae Lee

**Affiliations:** ^1^ Department of Life Science Research Institute for Natural Sciences Hanyang University Seoul 04763 Korea; ^2^ Department of Lymphoma and Myeloma Division of Cancer Medicine Center for Cancer Immunology Research The University of Texas MD Anderson Cancer Center Houston TX 77030 USA; ^3^ Laboratory of Radiation Exposure & Therapeutics National Radiation Emergency Medical Center Korea Institute of Radiological and Medical Sciences Seoul 01812 Korea; ^4^ Department of Neurosurgery Brain Tumor Center Severance Hospital Yonsei University College of Medicine Seoul 03722 Korea

**Keywords:** glioblastoma (GBM), macrophages, mesenchymal shift, soluble intercellular adhesion molecule‐1 (sICAM‐1), tumor microenvironment

## Abstract

Despite aggressive clinical treatment, recurrence of glioblastoma multiforme (GBM) is unavoidable, and the clinical outcome is still poor. A convincing explanation is the phenotypic transition of GBM cells upon aggressive treatment such as radiotherapy. However, the microenvironmental factors contributing to GBM recurrence after treatment remain unexplored. Here, it is shown that radiation‐treated GBM cells produce soluble intercellular adhesion molecule‐1 (sICAM‐1) which stimulates the infiltration of macrophages, consequently enriching the tumor microenvironment with inflammatory macrophages. Acting as a paracrine factor, tumor‐derived sICAM‐1 induces macrophages to secrete wingless‐type MMTV integration site family, member 3A (WNT3A), which promotes a mesenchymal shift of GBM cells. In addition, blockade of either sICAM‐1 or WNT3A diminishes the harmful effect of radiation on tumor progression. Collectively, the findings indicate that cellular crosstalk between GBM and macrophage through sICAM‐1‐WNT3A oncogenic route is involved in the mesenchymal shift of GBM cells after radiation, and suggest that radiotherapy combined with sICAM‐1 targeted inhibition would improve the clinical outcome of GBM patients.

## Graphical abstract

This study identifies the functional role of soluble intercellular adhesion molecule‐1 (sICAM‐1) for the mesenchymal shift of glioblastoma multiforme (GBM) in the tumor microenvironment. Importantly, the shedding of ICAM‐1 is increased in GBM following radiation and recruited macrophages for GBM progression. Taken together, the findings suggest sICAM‐1 as a molecular target to enhance the efficacy of radiotherapy.

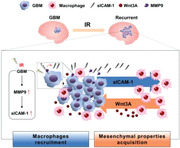

## 1 Introduction

Glioblastoma, also as known as glioblastoma multiforme (GBM) is the highest grade of glioma and the most aggressive type of cancer. For treatment of GBM, maximal surgical resection is followed by radiotherapy combined with temozolomide chemotherapy.^[^ ^1^ ^]^ Despite this aggressive treatment, recurrence is nearly inevitable, and the clinical benefit is marginal with a median survival time of only 15 months.^[^ ^1^ ^]^ In GBM subtypes molecularly defined based on clinical relevance, mesenchymal‐type exhibited greater invasiveness and worse prognosis compared to the other subtypes.^[^ ^2^ ^]^ Meanwhile, several strong lines of evidence suggest that radiation induces a mesenchymal shift of GBM.^[^ ^3^ ^]^ However, microenvironmental regulators contributing to mesenchymal shift and the consequent recurrence after radiation remain unexplored.

Intercellular adhesion molecule‐1 (ICAM‐1), a transmembrane glycoprotein belonging to the immunoglobulin superfamily of adhesion molecules, is well‐known in stabilizing cell–cell interaction and facilitating leukocyte‐endothelial transmigration.^[^ ^4^ ^]^ Moreover, a portion of ICAM‐1 is shed from the cell surface by proteolytic cleavage and possibly plays a role in both endocrine and paracrine manners.^[^ ^5^ ^]^ However, the clinical significance of ICAM‐1 remains controversial in cancer biology.^[^ ^6^ ^]^

Here, we demonstrate that soluble ICAM‐1 (sICAM‐1) is increased in GBM upon radiation. Importantly, sICAM‐1 recruits macrophages to the tumor microenvironment, and tumor‐educated macrophages produce wingless‐type MMTV integration site family, member 3A (WNT3A) for the mesenchymal shift of GBM. Collectively, our findings reveal that the oncogenic route between sICAM‐1 and WNT3A is important as cellular crosstalk is involved in the mesenchymal shift of GBM after radiation, suggesting that a targeted therapy of sICAM‐1 in combination with radiotherapy would be the beneficial therapeutic strategy of GBM treatment.

## 2 Results

### 2.1 Radiation‐Induced sICAM‐1 Promotes a Mesenchymal Shift of GBM Only In Vivo

**Figure 1:**
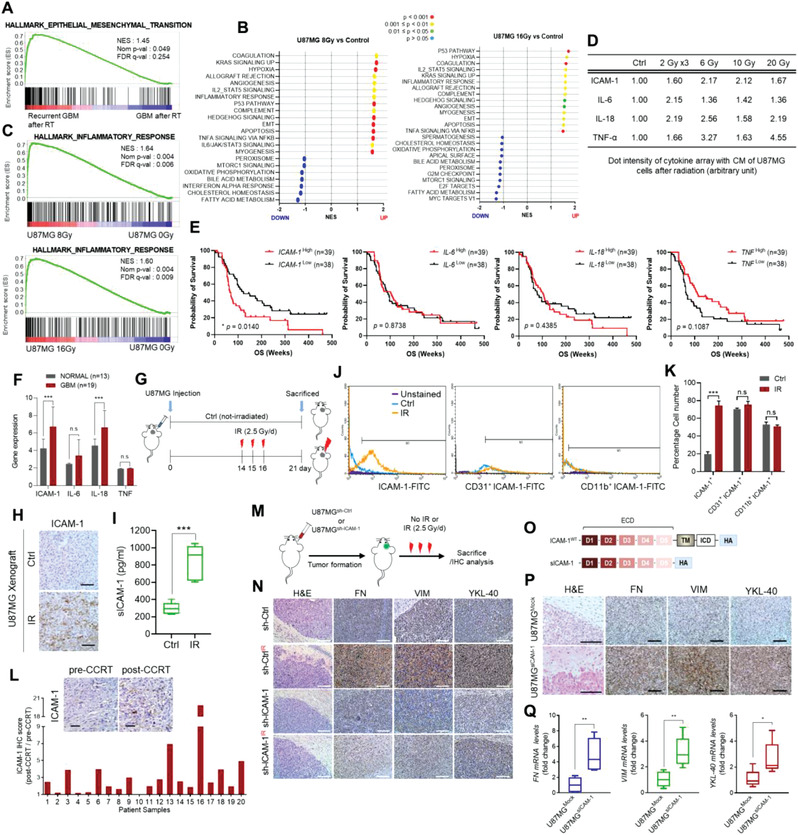
Radiation‐induced soluble ICAM‐1 promotes mesenchymal shift of GBM. A) Gene set enrichment analysis of GBM patients according to recurrence after radiation therapy (GSE7696). B,C) GSEA plots of normalized enrichment score (NES) of radiation exposure‐responsive gene set in human U87MG compared to non‐irradiated cells (GSE56937). D) Table summarizing the quantification of cytokines in the conditioned media of U87MG cells exposed to radiation with either a fractionated dose, 2Gy/d × 3 (2Gy per day for 3 days), or single dose (6, 10, or 20Gy). E) Overall survival curves for ICAM‐1, IL‐6, IL‐18, and TNF‐*α* in patients with GBM based on low and high expression (GSE4271, *n* = 77). F) Cytokine expression levels in normal human brain and GBM (GSE66354). G) Schematic of the animal experimental design. U87MG cells were injected intracranially into BALB/c nude mice (*n* = 6 per group). H) IHC analysis of ICAM‐1 and I) ELISA of sICAM‐1 level in U87MG orthotopic xenograft tumor in BALB/c nude mice after cranial irradiation. Scale bar, 200 µm. J,K) FACS analysis was performed using U87MG orthotopic xenograft tumors to assess ICAM‐1 expression in tumors, endothelial cells, and macrophages. L) IHC analysis and graphical depiction of the ratio of post‐CCRT to pre‐CCRT IHC intensity in specimens from patients with GBM. Scale bar, 100 µm. M) Schematic of the animal experimental design. U87MG^sh‐Ctrl^ or U87MG^sh‐ICAM‐1^ cells were injected orthotopically into BALB/c nude mice (*n* = 6 per group). N) H&E and IHC analysis of FN, VIM, and YKL‐40 in U87MG^sh‐Ctrl^ or U87MG^sh‐ICAM‐1^ orthotopic xenograft tumors after irradiation. Scale bar, 200 µm. O) Schematic diagram of expression constructs encoding ICAM‐1 wild type and soluble ICAM‐1. P) H&E and IHC analysis of FN, VIM, and YKL‐40 in U87MG^Mock^ and U87MG^sICAM‐1^ orthotopic tumors (*n* = 6 per group). Scale bar, 200 µm. Q) RT‐qPCR of FN, VIM, and YKL‐40 in U87MG^Mock^ and U87MG^sICAM‐1^ orthotopic tumors. Data are presented as mean ± SD. *β*‐actin was used as control for normalization of expression. n.s, non‐significant; *, *p* < 0.05 versus control; **, *p* < 0.01 versus control; ***, *p* < 0.001 versus control. A two‐tailed Student's *t*‐test was used to compare data between two groups.

GBM with recurrence after radiotherapy showed higher expression of the genes related to epithelial to mesenchymal transition (EMT) (GSE7696) (**Figure** **1**A). To investigate the transition of the genetic profile after radiation, we analyzed GBM dataset (GSE56937) and found that inflammatory signature genes are significantly induced (Figure 1B,C). Therefore, we performed a cytokine array after the various doses of radiation to define the radiation‐induced secretion factors. Notably, sICAM‐1, IL‐6, IL‐18, and TNF‐*α* were significantly induced in U87MG in response to the radiation (Figure 1D and Figure S1A, Supporting Information). In particular, sICAM‐1 was consistently induced by a broad spectrum of radiations in GBM cell lines, and patient‐derived X01.^[^ ^7^ ^]^ (Figure S1B–E, Supporting Information). In addition, compared with other soluble factors, ICAM‐1 was significantly correlated with a poor survival rate (GSE4271, GSE74187) and showed higher expression in GBM than in the normal brain (GSE66354) (Figure 1E,F and Figure S1F, Supporting Information).

Several strong lines of evidence showed that radiation enhances mesenchymal features of cancer cells,^[^ ^3^, ^8^ ^]^ and we confirmed that radiation promoted mesenchymal phenotypes in GBM (Figure S2A,B, Supporting Information). Given that sICAM‐1 was increased after radiation, the potential role of sICAM‐1 in the mesenchymal shift of GBM was examined. Next, we analyzed sICAM‐1 in U87MG‐orthotopic xenograft athymic BALB/c nude mice (*n* = 6 per group) and in GL261‐syngeneic C57BL/6 mice (*n* = 7 per group) (Figure 1G and Figure S2C, Supporting Information). When intracranial tumors were formed, tumors were irradiated locally with 2.5Gy/d × 3. Immunohistochemistry (IHC), ELISA and FACS analysis revealed that ICAM‐1 was increased in irradiated tumors (Figure 1H–K and Figure S2D–H, Supporting Information); however, an increase of ICAM‐1 was not observed in endothelial cells or macrophages which are known as mainly expressing ICAM‐1 (Figure 1J,K and Figure S2F–H, Supporting Information). Besides, the amount of sICAM‐1 was also upregulated in the blood extracted from irradiated mice. (Figure S2I, Supporting Information). Importantly, ICAM‐1 was relatively higher in the post‐concurrent chemoradiotherapy (CCRT) than the paired pre‐CCRT specimens (Figure 1L).

To this end, we transfected U87MG with sh‐ICAM‐1 and injected intracranially into BALB/c nude mice (*n* = 6 per group) (Figure 1M). Increased tumor infiltration and mesenchymal shift were observed in irradiated mice (Figure 1N); however, this was not the case in tumors formed by U87MG^sh‐ICAM‐1^. In addition, similar results were observed in GL261‐syngeneic model (*n* = 7 per group) (Figure S2J–L, Supporting Information). To confirm the role of sICAM‐1, we overexpressed sICAM‐1 in GBM cells and generated xenograft tumors (*n* = 6 per group for U87MG xenograft models, *n* = 7 per group for GL261 syngeneic model) (Figure 1O–Q and Figure S2M–R, Supporting Information). Similar to radiation, sICAM‐1 caused the mesenchymal shift and tumor dissemination from the margin in both xenograft tumors (Figure 1P,Q and Figure S2Q,R, Supporting Information); however, an effect on proliferation or apoptosis was not observed (Figure S2S,T, Supporting Information). In contrast, sICAM‐1 had no effect on invasion/migration, cell growth, and mesenchymal shift in vitro (Figure S2U–W, Supporting Information).

Taken together, these results show that radiation increases sICAM‐1 in GBM, and sICAM‐1 promotes the mesenchymal shift of GBM only in vivo but not in vitro. Therefore, we postulated that sICAM‐1 acts as a microenvironmental cue in GBM complexity.

### 2.2 sICAM‐1 is Generated by MMP‐9‐Catalyzed Proteolytic Cleavage of Membrane‐Bound ICAM‐1 upon Radiation

Based on the above data, radiation induces the de novo gene transcription of ICAM‐1, but sICAM‐1 can be produced by proteolytic cleavage of the membrane‐bound form of ICAM‐1 as well as alternative splicing of ICAM‐1 primary transcript.^[^ ^5^, ^9^ ^]^ To discriminate the radiation‐induced sICAM‐1 is due to only *de novo* gene transcription or affecting from shedding of pre‐existing membrane‐bound ICAM‐1, we examined the involvement of matrix metalloproteinases (MMPs) and a disintegrin and metalloproteinases, by treatment with each inhibitor. Notably, radiation‐induced sICAM‐1 was greatly affected by MMP9 (Figure S3A–C, Supporting Information). Consistent with previous reports,^[^ ^10^ ^]^ expression and activation of MMP‐9 were promoted by radiation (Figure S3D–F, Supporting Information). To confirm radiation‐induced cleavage of ICAM‐1 through MMP‐9, we transfected GBM cells with mutant ICAM‐1^P404E^ in which proline in position 404, the cleavage site in ICAM‐1, was substituted with glutamic acid (P404E) (Figure S3G,H, Supporting Information). Notably, MMP‐9 failed to shed ICAM‐1 in ICAM‐1^P404E^‐transfected GBM cells (Figure S3I, Supporting Information). In addition, transfection with ICAM‐1^WT^ alone increased sICAM‐1 marginally; however, co‐treatment with rh‐MMP‐9 increased sICAM‐1 drastically. To determine whether the increased level of sICAM‐1 is also a regulation at the transcriptional level as well as shedding (cleavage) of ICAM‐1 by protease, we examined secretion of sICAM‐1 in U87MG cells after pre‐treatment of Actinomycin D (ActD). ActD exhibited an inhibitory effect on irradiation (IR)‐induced sICAM‐1 secretion (Figure S3J,K, Supporting Information). However, seeing that the expression of ICAM‐1 and MMP9 was decreased by ActD as well (Figure S3L,M, Supporting Information). Accordingly, we examined treatment with rh‐MMP9 can recover the secretion of sICAM‐1. We showed that treatment with rh‐MMP9 can partially increase the secretion of sICAM‐1 after irradiation (Figure S3J–M, Supporting Information). These results suggested that both gene transcription and shedding of ICAM‐1 are induced by radiation. Next, we attempted to evaluate our findings in vivo by analyzing the mesenchymalization in U87MG subcutaneous xenograft in athymic BALB/c nude mice (*n* = 4 per group) after treatment with rh‐MMP9 (Figure S3N, Supporting Information). IHC analysis showed that mesenchymalization was partially increased in primary tumors formed by U87MG^ICAM‐1^ than U87MG^Ctrl^, and treatment with rh‐MMP9 had much accelerated its mesenchymal shift (Figure S3O, Supporting Information). Therefore, radiation treatment increases MMP‐9 expression which is able to generate soluble ICAM‐1 from the membrane‐bound ICAM‐1, which might have a significant contribution to the malignancy of irradiated tumors.

### 2.3 Radiation‐Induced sICAM‐1 Acts as a Chemoattractant for Macrophages Promoting the Mesenchymal Shift of GBM in the Tumor Microenvironment

**Figure 2:**
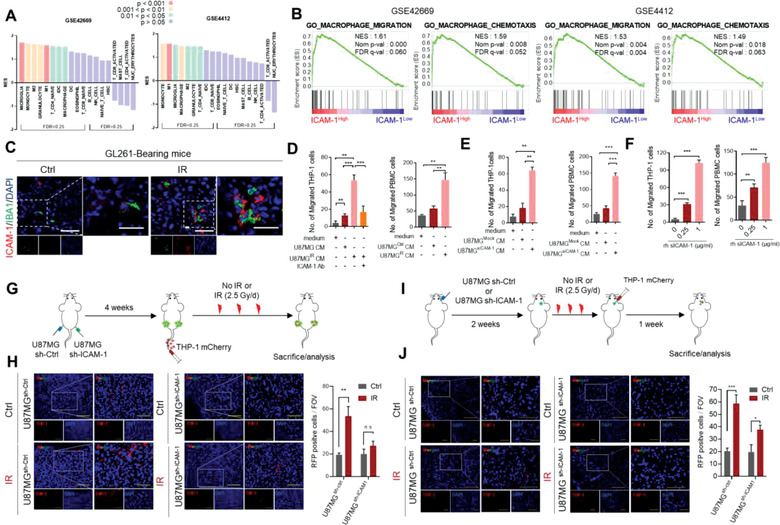
Radiation‐induced sICAM‐1 recruits bone marrow‐derived macrophages to the tumor, resulting in mesenchymal shift of GBM. A) GSEA for 17 types of immune cells in ICAM‐1^High^ and ICAM‐1^Low^ GBM patients from GEO dataset. B) GSEA plots for a significant correlation between high expression of ICAM‐1 and gene sets of macrophage migration and chemotaxis. C) IHC analysis for IBA‐1 and ICAM‐1 in GL261 orthotopic syngeneic tumors irradiated (2.5 Gy per day for 3 days) or not irradiated (*n* = 7 per group). Scale bar, 100 µm. D) Quantification of mobility of THP‐1 macrophages and PBMCs in CM of U87MG cells irradiated or non‐irradiated, and following treatment with ICAM‐1 antibody. E) Quantification of mobility of THP‐1 macrophages and PBMCs in CM of U87MG cells transfected with sICAM‐1 or mock construct. F) Effect of rh‐sICAM‐1 on mobility of THP‐1 macrophages and PBMCs. G) Schematic of the animal experiment design for preferential recruitment of THP‐1 macrophages to U87MG^sh‐Ctrl^ or U87MG^sh‐ICAM‐1^ xenograft tumors in BALB/c nude mice after irradiation (*n* = 3 per group). H) IHC analysis of mCherry‐labeled THP‐1 macrophages in U87MG^sh‐Ctrl^ or U87MG^sh‐ICAM‐1^ xenograft tumors after irradiation. Scale bar, 200 µm. I) Schematic of the animal experiment design for preferential recruitment of THP‐1 macrophages to U87MG^sh‐Ctrl^ or U87MG^sh‐ICAM‐1^ orthotopic xenograft tumors in BALB/c nude mice after irradiation (*n* = 3 per group). J) IHC analysis of mCherry‐labeled THP‐1 macrophages in orthotopic xenograft mice. Scale bar, 200 µm. Data are presented as mean ± SD. *β*‐actin was used as control for normalization of expression. n.s, non‐significant; *, *p* < 0.05 versus control; **, *p* < 0.01 versus control; ***, *p* < 0.001 versus control. A two‐tailed Student's *t*‐test was used to compare data between two groups.

A variety of immune cells are recruited into the tumor microenvironment, and this event is more severe in irradiated tumors.^[^ ^11^ ^]^ Therefore, we examined GBM datasets for 17 types of immune cells gene signatures^[^ ^12^ ^]^ to discover the specific immune cells that are recruited by sICAM‐1. These analyses revealed that the high expression of ICAM‐1 is correlated with the enrichment of macrophage‐signature genes (**Figure** **2**A). Given that LFA‐1 and MAC‐1, receptors for ICAM‐1, are mainly expressed in macrophages, we hypothesized sICAM‐1 could act as a chemoattractant for macrophages toward the tumor microenvironment. Notably, gene set enrichment analysis (GSEA) showed that high expression of ICAM‐1 was associated with macrophage recruitment‐signature genes (Figure 2B). In the GL261 syngeneic model, we noticed a concurrent increase of sICAM‐1 and macrophages (IBA‐1^+^)^[^ ^13^ ^]^ after radiation (*n* = 7 per group) (Figure 2C). In addition, sICAM‐1 overexpression also increased the number of IBA‐1^+^ and CD11b^+^ macrophages (*n* = 7 per group) (Figure S4A, Supporting Information). Next, we observed that C‐C chemokine receptor type 2 (CCR2)^+^ cells were markedly increased than CX3C chemokine receptor 1 (CX_3_CR_1_)^+^ cells in syngeneic tumors in response to radiation or sICAM‐1 overexpression (Figure S4B–E, Supporting Information). CCR2 is a marker for bone marrow‐derived macrophages, whereas CX_3_CR_1_ is a marker for resident brain microglia.^[^ ^14^ ^]^ We thus speculated that radiation‐induced sICAM‐1 contributes to the recruitment of bone marrow‐derived macrophages. We also analyzed the mobility of THP‐1 and peripheral blood mononuclear cells (PBMCs) after treatment with conditioned medium (CM) of irradiated or non‐irradiated U87MG. THP‐1 which have known as a model for human monocyte and monocyte‐macrophage differentiation^[^ ^13^ ^]^ were differentiated using phorbol 12‐myristate 13‐acetate (PMA) into macrophage‐like cells. Importantly, migration of THP‐1 and PBMC were significantly increased after treatment with CM from irradiated GBM cells and diminished by blocking ICAM‐1 (Figure 2D and Figure S4F–H, Supporting Information). Furthermore, THP‐1 and PBMC mobility were increased by treatment with CM of sICAM‐1‐overexpressing GBM cells or rh‐sICAM‐1 (Figure 2E,F and Figure S4I, Supporting Information).

As sICAM‐1 promoted mesenchymal shift of GBM only in vivo, we examined whether sICAM‐1 provokes mesenchymal shift of GBM by macrophage recruitment. To this end, we cocultured GBM cells with THP‐1. Although radiation alone promoted invasion and mesenchymal marker expression of GBM, THP‐1‐coculture synergistically added up to the effect of radiation (Figure S4J–L, Supporting Information). However, coculture with THP‐1 had no significant effect on the invasion of non‐irradiated GBM cells. Furthermore, treatment with si‐ICAM‐1 or MMP‐9 inhibitor diminished the synergistic effect of radiation and THP‐1‐coculture (Figure S4M, Supporting Information). In agreement with these observations, sICAM‐1‐induced mesenchymal shift of GBM only in coculture with THP‐1 (Figure S4N, Supporting Information).

Next, we introduced mCherry‐labeled THP‐1 into the tail vein of mice carrying subcutaneous xenograft tumors (*n* = 3 per group) (Figure 2G). The recruitment of THP‐1^mCherry^ into xenograft tumors was increased following radiation (2.5Gy/d × 3); however, the effect of radiation was not observed in tumors formed by U87MG^sh‐ICAM‐1^ (Figure 2H). To further examine the recruitment of macrophages to GBM microenvironment, both U87MG and THP‐1^mCherrpy^ were injected intracranially into the athymic nude mice (*n* = 3 per group) (Figure 2I). Importantly, the number of recruited THP‐1 into xenograft tumors was increased by radiation (2.5Gy/d × 3); however, ICAM‐1 depletion in U87MG attenuated the effect of radiation (Figure 2J). To evaluate the role of sICAM‐1 in macrophage recruitment in vivo, U87MG^sICAM‐1^ were inoculated subcutaneously or orthotopically and THP‐1^mCherry^ were injected into the tail veins or into the cranium respectively. In both xenograft models, THP‐1^mCherry^ were more recruited into U87MG^sICAM‐1^ tumors (*n* = 3 per group) (Figure S4O–R, Supporting Information) and U87MG^sICAM‐1^ tumors highly expressed mesenchymal markers (Figure S4S, Supporting Information). Taken together, these results demonstrate that sICAM‐1 acts as a chemoattractant for macrophages into the tumor microenvironment, thereby promoting the mesenchymal shift of GBM.

### 2.4 Radiation‐Induced sICAM‐1 Promotes WNT3A Secretion in Macrophages, a Signaling Cue for the Mesenchymal Shift of GBM in the Tumor Microenvironment

**Figure 3:**
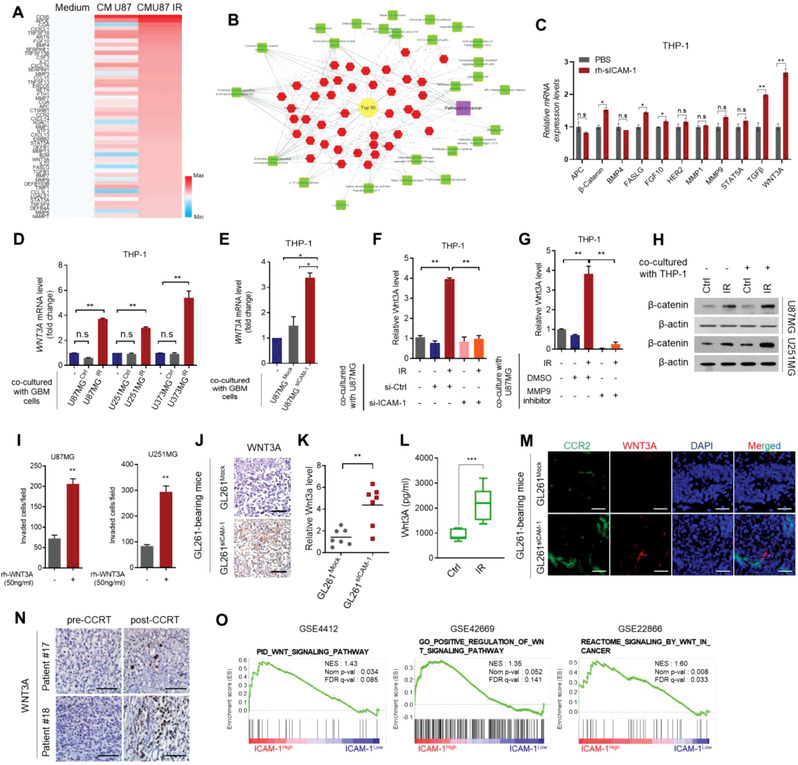
Radiation‐induced sICAM‐1 promotes WNT3A secretion by bone marrow‐derived macrophages after recruitment to GBM site. A) Top 50 list of cytokine profiling antibody array for THP‐1 macrophages after treatment with control medium or CM of U87MG cells that are irradiated or non‐irradiated (detailed in Table S1, Supporting Information). B) Analysis of cytokines induced by CM of irradiated U87MG cells. The network was built based on the KEGG pathway. C) RT‐qPCR analysis of soluble factor expression induced in THP‐1 macrophages after treatment with rh‐sICAM‐1. D) RT‐qPCR analysis of WNT3A expression in THP‐1 macrophages cocultured with irradiated or non‐irradiated GBM cells. E) RT‐qPCR analysis of WNT3A expression in THP‐1 macrophages cocultured with U87MG^Mock^ or U87MG^sICAM‐1^ cells. RT‐qPCR analysis of WNT3A expression in THP‐1 macrophages F) when cocultured with U87MG GBM cells transfected with ICAM‐1 siRNA or G) treated with MMP‐9 inhibitors prior to irradiation. H) Western blot analysis of *β*‐catenin in U87MG or U251MG cells alone or cocultured with THP‐1 macrophages. I) Effect of rh‐WNT3A on migration of GBM cells in Transwell plates. J) IHC analysis of WNT3A in GL261^Mock^ or GL261^sICAM‐1^ orthotopic syngeneic tumor (*n* = 7 per group). Scale bar, 200 µm. K) RT‐qPCR analysis of WNT3A expression in GL261^Mock^ or GL261^sICAM‐1^ orthotopic syngeneic tumor. L) The amount of WNT3A in blood extracted from Heart in GL261‐bearing syngeneic mice. M) IHC analysis of CCR2 and WNT3A in GL261^Mock^ or GL261^sICAM‐1^ orthotopic syngeneic tumor. Scale bar, 100 µm. N) IHC analysis of WNT3A levels in a pair of specimens of GBM patient before CCRT (pre‐CCRT) and post‐CCRT. Scale bar, 200 µm. O) GSEA plots (GSE4412, 42669, and 22866) for a significant correlation between high expression of ICAM‐1 and WNT signaling. Data are presented as mean ± SD. *β*‐actin was used as control for normalization of expression. n.s, non‐significant; *, *p* < 0.05 versus control; **, *p* < 0.01 versus control; ***, *p* < 0.001 versus control. A two‐tailed Student's *t*‐test was used to compare data between two groups.

We next investigated how recruited macrophages influence GBM to transition into mesenchymal‐type. Multiple previous studies demonstrated that the immune cells that are recruited into the tumor microenvironments are educated after interacting with tumors and promote cancer malignancy.^[^ ^15^ ^]^ Given that, we expected tumor‐derived sICAM‐1 may educate recruited macrophages to affect the cytokine profile. To prove our hypothesis, we examined the secretion factors in THP‐1 after treatment with CM from U87MG that were irradiated or not by using a cytokine profiling array and analyzed the top 50 cytokines based on KEGG pathway. (**Figure** **3**A,B and Table S1, Supporting Information). Among these candidates, 12 secretion factors were belonging to the pathway in cancer. Therefore, we examined the expression of these factors after treatment with rh‐sICAM‐1 and found that WNT3A was the most upregulated (Figure 3C). In concordance with these results, WNT3A expression was increased in THP‐1 after cocultured with irradiated GBM (Figure 3D) which is consistent with THP‐1 with GBM cells overexpressing sICAM‐1 (Figure 3E). However, when sICAM‐1 was blocked, induction of WNT3A was not observed (Figure 3F,G). As Wnt3A is one of the canonical Wnt pathways, we analyzed *β*‐catenin in GBM cells after coculture with THP‐1. Interestingly, although *β*‐catenin and its target genes including AXIN2, LEF1, and MYC were increased in GBM cells by radiation alone, the effect of radiation was certainly enhanced by coculture with THP‐1 (Figure 3H, Figure S5A, Supporting Information). However, the expression of FZD1 and LRP1 which are known as WNT receptors was not changed by radiation (Figure S5B, Supporting Information). Similar effects were observed after rh‐WNT3A treatment (Figure S5C–F, Supporting Information).

We next examined whether WNT3A contributes to the mesenchymal shift of GBM. Treatment with rh‐WNT3A enhanced invasion of GBM and induced mesenchymal markers (Figure 3I and Figure S5G, Supporting Information); however, an effect on tumor growth was not observed (*n* = 4 per group) (Figure S5H–K, Supporting Information). WNT3A was also significantly elevated in tumors formed by GL261^sICAM‐1^ (Figure 3J,K). Besides, the amount of WNT3A was highly increased as well in the blood of sICAM‐1‐overexpressing group (Figure 3L). By co‐localization analysis, the number of co‐localized CCR2^+^ macrophages and Wnt3A was increased in xenograft tumors formed by GL261^sICAM‐1^ (Figure 3M). Consistently, WNT3A was higher with IBA‐1^+^ macrophages in post‐CCRT compared to paired pre‐CCRT (Figure 3N and Figure S5L, Supporting Information). GSEA revealed ICAM‐1 expression was also correlated with WNT signaling signature genes in GBM (Figure 3O). Collectively, these results suggest that radiation‐induced sICAM‐1 promotes WNT3A secretion from macrophages enables phenotypic change of GBM into the mesenchymal state.

### 2.5 sICAM‐1 Binds to LFA‐1 on Macrophages for Its Recruitment and WNT3A Signaling Leading to the Mesenchymal Shift of GBM

Given that LFA‐1 (CD11a/CD18) and Mac‐1 (CD11b/CD18) interact with ICAM‐1,^[^ ^16^ ^]^ we attempted to block the potential binding with ligands. When we blocked LFA‐1 or Mac‐1, THP‐1 mobility was no longer enhanced by treatment with U87MG^sICAM‐1^CM (Figure S6A, Supporting Information). Accordingly, because extracellular domain‐1 and domain‐3 of ICAM‐1 are known as binding sites for LFA‐1 and Mac‐1, respectively, we made expression constructs encoding ICAM‐1^ΔD1^ or ICAM‐1^ΔD3^ (Figure S6B,C, Supporting Information). Even though these deletion mutants were cleavable by MMP‐9, they failed to increase the mobility of THP‐1 (Figure S6D,E, Supporting Information). Collectively, these results demonstrate that sICAM‐1 stimulates the migration of macrophages through interaction with LFA‐1 and Mac‐1.

We next examined whether LFA‐1 and Mac‐1 were involved in WNT3A secretion. Treatment with CD11a antibody, which blocks LFA‐1 binding, dissipated the induction of WNT3A by coculture with U87MG^IR^, whereas blocking Mac‐1 binding with CD11b antibody could not attenuate this effect (Figure S6F, Supporting Information). Besides, CD11a antibody also attenuated invasion of GBM that was enhanced by treatment with rh‐sICAM‐1 in coculture with THP‐1 (Figure S6G, Supporting Information). However, CD11b antibody could not disturb the effect of rh‐sICAM‐1 in THP‐1 coculture on the invasion of GBM, indicating that interaction of sICAM‐1 with LFA‐1 is critical for induction of WNT3A in macrophages, whereas Mac‐1 is not involved in this event. Moreover, we examined whether blocking LFA‐1 could also diminish the mesenchymal shift of GBM. In agreement with the above data, coculture with THP‐1 induced mesenchymal markers along with *β*‐catenin in U87MG^sICAM‐1^; however, treatment with CD11a antibody significantly diminished this effect (Figure S6H, Supporting Information). By contrast, treatment with CD11b antibody failed to dissipate the effect on the mesenchymal shift of GBM (Figure S6I, Supporting Information).

Next, we cocultured THP‐1 with U87MG transfected with ICAM‐1 constructs following rh‐MMP‐9 treatment. Notably, WNT3A was higher in THP‐1 cocultured with U87MG^ICAM‐1‐ΔD3^ or U87MG^ICAM‐1‐WT^ (Figure S6J, Supporting Information). However, WNT3A was not increased in THP‐1 cocultured with U87MG^ICAM‐1‐ΔD1^ or U87MG^ICAM‐1‐P404E^, even by treatment with rh‐MMP‐9.

Taken together, these results suggest that extracellular domain‐1 in sICAM‐1 is critical for both macrophage recruitment and WNT3A secretion by macrophages, as well as the consequent mesenchymal shift of GBM. Whereas extracellular domain‐3 is only involved in macrophage recruitment.

### 2.6 Blocking sICAM‐1 or WNT3A Inhibits the Mesenchymal Shift and Infiltration of GBM by Suppressing Macrophages Recruitment

**Figure 4:**
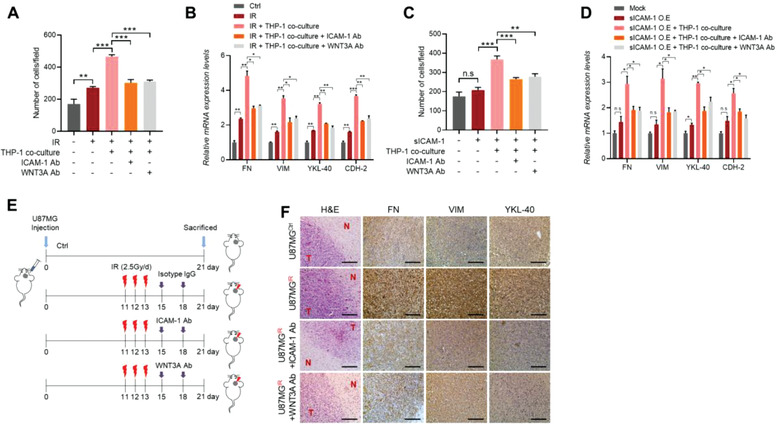
Blocking sICAM‐1 or WNT3A inhibits the infiltration and mesenchymal shift of GBM by suppressing macrophages recruitment. A) Invasion assay of U87MG cells cocultured with THP‐1 in combination treatments with radiation and/or a neutralizing antibody against ICAM‐1 or WNT3A as indicated. B) RT‐qPCR analysis of mesenchymal markers in U87MG cells cocultured with THP‐1 in combination treatments with radiation and/or a neutralizing antibody against ICAM‐1 or WNT3A. C) Invasion assay of U87MG cells cocultured with THP‐1 in combination treatments with sICAM‐1 transfection and/or a neutralizing antibody against ICAM‐1 or WNT3A as indicated. D) RT‐qPCR analysis of mesenchymal markers in U87MG cells cocultured with THP‐1 in combination treatments with sICAM‐1 transfection and/or a neutralizing antibody against ICAM‐1 or WNT3A as indicated. E) Schematic of the animal experiment design for evaluating therapeutic effect of neutralizing antibody against ICAM‐1 or WNT3A in U87MG xenograft BALB/c nude mice model (*n* = 5 per group). F) H&E and IHC analysis of FN, VIM, and YKL‐40 in U87MG orthotopic xenograft tumors after combination treatment with irradiation and ICAM‐1 or WNT3A neutralizing antibody. Scale bar, 200 µm. Data are presented as mean ± SD. *β*‐actin was used as control for normalization of expression. n.s, non‐significant; *, *p* < 0.05 versus control; **, *p* < 0.01 versus control; ***, *p* < 0.001 versus control. A two‐tailed Student's *t*‐test was used to compare data between two groups.

To assess the therapeutic potential of blocking sICAM‐1 or WNT3A for the mesenchymal shift and infiltration of GBM, we treated either ICAM‐1 or WNT3A neutralizing antibody to irradiated U87MG cells which were cocultured with THP‐1. After blocked ICAM‐1 or WNT3A, the invasive ability and mesenchymal markers expression of GBM cells enhanced by THP‐1 co‐culture were diminished (**Figure** **4**A,B). Similar results were observed in U87MG cells transfected with sICAM‐1 (Figure 4C,D). Next, we examined the therapeutic effects of blocking sICAM‐1 or WNT3A in U87MG‐orthotopic xenograft athymic BALB/c nude mice (*n* = 5 per group) (Figure 4E). Intracranial tumors were irradiated locally with 2.5Gy/d × 3 and neutralizing antibodies against ICAM‐1 or WNT3A were introduced to the tail vein of mice. IHC analysis revealed that the infiltration and mesenchymal shift of xenograft tumors were augmented in the mouse group treated with irradiation and these increases were not observed in the mouse group treated with ICAM‐1 or WNT3A neutralizing antibody (Figure 4F). Taken together, these results indicate that blocking sICAM‐1 or WNT3A inhibits the mesenchimalization and invasiveness ability of irradiated GBM cells.

### 2.7 Clinical Importance of Bone Marrow‐Derived Macrophage Recruitment and WNT3A in Patients with GBM in Response to Radiotherapy‐Induced sICAM‐1

**Figure 5:**
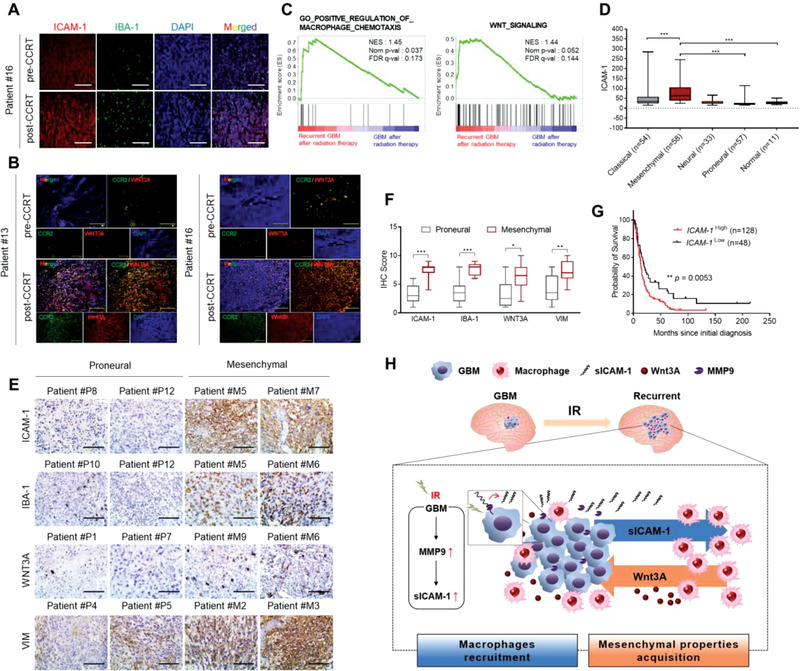
Increase in sICAM‐1 in patients with GBM after radiotherapy correlates with bone marrow‐derived macrophage recruitment, mesenchymal shift of tumors, and high levels of WNT3A. IHC analysis of A) ICAM‐1 and IBA‐1‐expressing cells or B) CCR2 expressing cells of GBM specimens from patients #13 and #16 pre‐CCRT and post‐CCRT. Scale bar, 200 µm. C) GSEA plots for correlation between gene sets of recurrent GBM after radiation and gene sets of macrophage chemotaxis or WNT signaling (GSE7696). D) ICAM‐1 expression levels in four different human GBM subtypes and normal human brain queried from the TCGA database. E) IHC analysis of ICAM‐1, IBA‐1, WNT3A, and VIM levels in GBM proneural and mesenchymal subtypes from patients. Scale bar, 200 µm. F) Quantification of IHC analysis in (E). G) Kaplan‐Meier survival curves of patients with GBM using Rembrandt database; ICAM‐1^High^ versus ICAM‐1^Low^. H) Schematic summarizing a model of mesenchymal shift of GBM cells caused by WNT3A signaling in tumor microenvironment triggered by a crosstalk between bone marrow‐derived macrophages and GBM cells in response to radiation‐induced sICAM‐1. Data are presented as mean ± SD. *β*‐actin was used as control for normalization of expression. n.s, non‐significant; *, *p* < 0.05 versus control; **, *p* < 0.01 versus control; ***, *p* < 0.001 versus control. A two‐tailed Student's *t*‐test was used to compare data between two groups.

To confirm clinical relevance‐regarding sICAM‐1 in the mesenchymal transition of GBM following radiation, we analyzed the extent of macrophage density in a pair of pre‐CCRT and post‐CCRT GBM specimens. The density of IBA‐1^+^CCR2^+^ macrophages was significantly greater in post‐CCRT than in pre‐CCRT (**Figure** **5**A,B). Given that sICAM‐1 promoted mesenchymalization of GBM, we analyzed GEO dataset and the cancer genome atlas project (TCGA). GSEA revealed that GBM with recurrence after radiotherapy has higher expression of macrophage chemotaxis and WNT signaling signature genes (Figure 5C). TCGA GBM showed that ICAM‐1 was overrepresented in mesenchymal‐type and underrepresented in proneural‐type (Figure 5D). Moreover, IBA‐1, WNT3A, and ICAM‐1 were higher in mesenchymal‐type, suggesting that mesenchymalization of GBM is correlated with macrophage enrichment, and high expression of WNT3A and ICAM‐1 (Figure 5E,F). We finally examined the survival rate of GBM patients with ICAM‐1 expression. Using the Rembrandt database, patients with high expression of ICAM‐1 showed shorter survival than the rest of the patients (Figure 5G). Taken together, these results suggest that sICAM‐1, which is elevated upon radiation, acts as a chemoattractant that entices macrophages into tumor‐microenvironment and stimulates these macrophages to secrete WNT3A, which in turn induces the transition of GBM into the mesenchymal state (Figure 5H).

## 3 Discussion

Much of the understanding of GBM in response to radiation comes from studies on glioblastoma cells cultured in vitro. However, GBM is more than an insular mass of proliferating tumor cells; the tumor microenvironment consisting of a variety of cytokines, extracellular matrix, and a repertoire of recruited non‐neoplastic cells forms a systemic and molecular network.^[^ ^17^ ^]^

In this study, we discover that the expression and secretion of ICAM‐1 are induced by radiation and it promotes GBM progression by regulating the tumor microenvironment. Elevation of sICAM‐1 is commonly observed in several pathological conditions, including multiple sclerosis, immune disorders, and cancer.^[^ ^18^ ^]^ Here we showed that sICAM‐1 was increased in GBM after radiation and it was able to detect in the blood of mice (Figure S2I, Supporting Information). This result implied sICAM‐1 as a potential diagnostic marker for malignant GBM.

Of note, sICAM‐1 had no direct effect on the mesenchymal shift of GBM; rather, it promoted this process by acting as a chemoattractant to recruit macrophages. sICAM‐1 recruits bone marrow‐derived macrophages rather than resident‐microglia and blockade of LFA‐1 or Mac‐1 on macrophages attenuated sICAM‐1‐dependent macrophage recruitment. Intriguingly, sICAM‐1 stimulated macrophages to produce WNT3A through LFA‐1 but not Mac‐1. Previously, WNT signaling is reported for aberrantly activation in GBM and promoting tumor growth and invasion.^[^ ^19^ ^]^ Here, we describe for the first time that high levels of WNT3A in GBM microenvironment are derived from macrophages that are recruited by sICAM‐1.

In summary, our findings show that sICAM‐1, which increased upon radiation, played a central role in the crosstalk between tumors and macrophages. Moreover, this study describes WNT3A as a critical regulator in the mesenchymal shift of GBM that occurred after radiation, suggesting that WNT3A could be a novel therapeutic target enhancing the efficacy of radiotherapy. As radiation is widely used for the treatment of patients with GBM, understanding the oncogenic signaling route between tumors and macrophages after radiation will provide potential therapeutic targeting to prevent malignancy in post‐irradiated GBM.

## 4 Conclusion

This study identified the functional role of sICAM‐1 for the mesenchymal shift of GBM in the tumor microenvironment. Importantly, the shedding of ICAM‐1 was increased in GBM following radiation and recruited macrophages for GBM progression. Intriguingly, blocking sICAM‐1 suppressed the mesenchymal shift of GBM. Taken together, our findings demonstrated that the critical role of sICAM‐1 in the cellular crosstalk in the tumor microenvironments, suggesting sICAM‐1 as a molecular target to enhance the efficacy of radiotherapy.

## 5 Experimental Section

### Cells

U87MG, U373MG, and U251MG glioma cells were obtained from the Korean Cell Line Bank (Seoul, Korea). Patient‐derived X01 cells were established from acutely resected human tumor tissues obtained with written informed consent from a 68‐year‐old woman with GBM and provided by Dr. Akio Soeda.^[^ ^7^ ^]^ GL261 mouse GBM cells were obtained from Yonsei Univ. U87MG, U373MG, U251MG, and patient‐derived X01 were cultured in Dulbecco's modified Eagle Medium (Invitrogen, Carlsbad, CA, USA) supplemented with 10% fetal bovine serum (FBS). GL261 were cultured in DMEM/F12 supplemented with 10% FBS. PBMCs were purchased from Zenbio. THP‐1 monocytes were purchased from Korean Cell Line Bank. THP‐1 were differentiated with 200 ng mL^−1^ PMA for 24 h into macrophage‐like cells, and were cultured in RPMI supplemented with 10% FBS.

### Chemical Reagents and Antibodies

MMP Inhibitor V and MMP Inhibitor Set I were purchased from Calbiochem (San Diego, CA, USA). GI254023X was purchased from Sigma‐Aldrich (St Louis, MO, USA). rh‐TNF‐*α* and rh‐WNT3A were purchased from R&D (Minneapolis, MN, USA), and rh‐sICAM‐1 was purchased from eBioscience (Waltham, MA, USA.). rhMMP‐9 and an antibody against WNT3A were purchased from Millipore (Billerica, MA, USA). Antibodies against HA‐tag, FN, ICAM‐1, anti‐mouse IgG‐HRP, anti‐goat IgG‐HRP, and anti‐rabbit IgG‐HRP were purchased from Santa Cruz Biotechnology (Santa Cruz, CA, USA). Antibodies to anti‐goat Alexa Fluor 488, anti‐mouse Alexa Fluor 546 were purchased from Invitrogen (Carlsbad, CA, USA). Antibodies against CD31, VIM, YKL‐40, p‐Src (Tyr418), IBA‐1, TNF‐*α*, CD11a, and ICAM‐1 were purchased from Abcam (Cambridge, UK). Antibodies against p‐PKC*δ* (Tyr311) and p‐STAT3 (Tyr705) were purchased from Cell Signaling Technology (Beverly, MA, USA). Antibodies against IBA‐1 (Novus Biologicals, Littleton, CO, USA), CD11b (BioLegend, San Diego, CA, USA), and N‐cadherin (BD Biosciences, San Jose, CA, USA) were also used in this study. Antibodies against *β*‐actin and ZEB1 were obtained from Sigma‐Aldrich.

### Human GBM Specimens

Tissues from patients with GBM were obtained from the Severance Hospital, Yonsei University, Seoul, Korea. GBM tissues were randomly collected from 20 patients who were diagnosed with GBM between 2009 and 2014. and had surgery, followed by treatment with either radiotherapy (1 case) or CCRT (19 cases) prior to relapse. GBM tissues were also obtained from 22 patients who were diagnosed with GBM between 2013 and 2016, and categorized into proneural or mesenchymal subtypes based on Single‐sample Gene Set Enrichment Score analysis from the normalized microarray data (Illumina HT12 v4). This study was approved by the Institutional Review Boards of Severance Hospital, Yonsei University College of Medicine (4‐2012‐0212). Informed consent was obtained from all patients in accordance with the Declaration of Helsinki. Neuropathologists diagnosed each surgical specimen according to World Health Organization (WHO) classifications.^[^ ^20^ ^]^

### GSEA and Dataset Evaluation

GSEA was performed on diverse gene signatures by comparing gene sets from either the Molecular Signature Database (MSigDB) database or published gene signatures. To analyze the expression profiles in glioblastoma with radiotherapy, GEO dataset (GSE56937, GSE7696, GSE4271, GSE66354, GSE4412, GSE42669, GSE22866, GSE74187), TCGA, and Rembrandt datasets were analyzed.

### Animal Experiments

Male athymic nude mice (Central Lab Animal Inc., Seoul, Korea), aged 6 to 8 weeks, were used in this study. U87MG GBM cells were implanted into the right frontal lobe of nude mice using a guide‐screw system within the skull, as described previously.^[^ ^21^ ^]^ A total of 2 × 10^5^ U87MG GBM cells were injected into each mouse using a multiple micro‐infusion syringe pump (Harvard Apparatus, Holliston, MA, USA) at a speed of 0.5 µL min^−1^. Mice with a similar weight and age were randomized into two experimental groups (*n* = 6 per group) (Figure 1G–K, M–Q and Figure S2S, Supporting Information). In the syngeneic model, C57BL/6 mice, aged 6 to 8 weeks, were used and total of 1.5 × 10^5^ mouse GL261 GBM cells were injected into each mouse (*n* = 7 per group) (Figures 2C, 3J–M and Figures S2 and S4, Supporting Information). Next procedure was the same as xenograft model. For THP‐1 recruitment assay in vivo, U87MG cells (1 × 10^6^) transfected with either sh‐Ctrl or sh‐ICAM‐1 were injected into the right and left flank of athymic nude mice (*n* = 3 per group) (Figure 2G,H). After 4 weeks, when xenograft tumors were formed, mCherry‐labeled THP‐1 macrophages were injected into the tail vein and tumors were locally irradiated. In another model, U87MG cells (2 × 10^5^) transfected with either sh‐Ctrl or sh‐ICAM‐1 were also injected orthotopically into mouse brains as described above (*n* = 3 per group) (Figure 2I,J). 2 weeks after inoculation of GBM cells, tumors were locally irradiated and mCherry‐labeled THP‐1 macrophages were intracranially injected into the mice, which were sacrificed for IHC analysis after 1 week. In addition, U87MG cells (1 × 10^6^) were transfected with a mock or sICAM‐1 expression vector prior to injection into the right and left flank of athymic nude mice (*n* = 3 per group) (Figure S4O,P, Supporting Information). After 4 weeks, when xenograft tumors were formed, mCherry‐labeled THP‐1 macrophages were injected into the tail vein. In another model, U87MG GBM cells (2 × 10^5^) transfected with either a mock or sICAM‐1 expression vector were also injected orthotopically into mouse brains as described above (*n* = 3 per group) (Figure S4Q–S, Supporting Information). 1 week after inoculation of GBM cells, mCherry‐labeled THP‐1 macrophages were intracranially injected into the mice, which were sacrificed for IHC analysis after 2 weeks. Tumor tissues were stained with anti‐mCherry antibody to detect recruited THP‐1 macrophages into xenograft tumor sites and quantified by counting the number of red cells in three randomly‐selected microscopic fields of xenograft tumor paraffin sections. To validate the mesenchymal shift effect of MMP9, U87MG cells (5 × 10^5^) were injected subcutaneously into athymic nude mice (*n* = 4 per group) (Figure S3N,O, Supporting Information). After 1 week, when xenograft tumors were formed, rh‐MMP9 (100 µg kg^−1^) was three times injected into the tumor site. To examine the tumorigenesis effect of WNT3A, GL261 cells (5 × 10^5^) were injected subcutaneously into C57BL/6 mice (*n* = 4 per group) (Figure S5H–K, Supporting Information). After 1 week, when xenograft tumors were formed, rm‐WNT3A (40 µg kg^−1^) was three times injected into the tumor site. Tumor growth and weight were measured after tumor formation. To validate the therapeutic effect of neutralizing antibodies against ICAM‐1 or WNT3A, U87MG cells were injected orthotopically into athymic nude mice (*n* = 5 per group) (Figure 4E,F). After 10 days, tumors were locally irradiated and neutralizing antibodies (3mg kg^−1^) were injected into the tail vein two times. All animal experiments were performed by following the guidelines of NC3Rs of (National Center for the Replacement, Refinement, and Reduction in Animal Research) and approved by the Institutional Animal Care and Use Committee, Severance Hospital, Yonsei University College of Medicine, Seoul, Korea.

### Western Blot Analysis

The membrane was blocked with 5% non‐fat dry milk in Tris‐buffered saline and incubated with primary antibodies overnight at 4 °C. Blots were developed with a peroxidase‐conjugated secondary antibody, and proteins were visualized by enhanced chemiluminescence (Amersham), according to the manufacturer's protocol. Cell lysates were prepared by extracting proteins with a lysis buffer [40 mm Tris–HCl (pH 8.0), 120 mm NaCl, 0.1% Nonidet‐P40] supplemented with protease inhibitors. Proteins were separated by SDS‐PAGE and transferred to a nitrocellulose membrane (Amersham, Arlington Heights, IL, USA).

### RT‐qPCR

Reactions were carried out in a Rotor Gene Q (Qiagene, Hilden, Germany) PCR cycler, and results were expressed as fold difference calculated by using the ΔΔCt method relative to the control sample. GAPDH was used as an internal normalization control.

### Migration and Invasion Assays

For invasion assay, GBM cells were loaded in Transwell plates with 8‐µm pore size filter inserts (Corning Glass, Seoul, Korea) that were precoated with 10 mg mL^−1^ growth factor‐reduced Matrigel (BD Biosciences, Seoul, Korea) on the upper side of the chamber with the lower well filled with 0.8 mL of growth medium. To analyze the effect of THP‐1 coculture on invasion of GBM cells, THP‐1 macrophages were seeded in lower wells. After incubation for 48 h at 37 °C, non‐invaded cells in the upper surface of the filter were removed with a cotton swab, and migrated cells on the lower surface of the filter were fixed and stained with the Diff‐Quick kit (Fisher Scientific, Pittsburgh, PA, USA), and observed (magnification ×20). Invasiveness was determined by counting cells in five microscopic fields per well, and the extent of invasion was expressed as an average number of cells per microscopic field. Cells were imaged by phase contrast microscopy (Leica Microsystems, Bannockburn, IL, USA). For migration assay, Transwell plates with inserts that contained the same type of membrane were used without Matrigel coating.

### Indirect Co‐Culture System

For co‐culture assay, PMA‐primed THP‐1 macrophage was loaded in the upper/lower well of 6well‐transwell plate (Corning). GBM cells were seeded in the lower/upper well. After incubation for 48 h at 37 °C, the cells in lower well were havested to extract RNA or protein.

### THP‐1 Mobility Assay

For recruitment assay, PMA‐primed THP‐1 macrophages were loaded in the upper well of Transwell plates with 8‐µm pore size filter inserts (Corning Glass) that were pre‐coated with 0.2% gelatin on the lower side of the chamber. U87MG GBM cells were seeded in the lower well, or it was filled with U87MG conditioned medium. After incubation for 48–72 h at 37 °C, non‐invading cells on the upper surface of the filter were removed with a cotton swab, and migrating THP‐1 macrophages on the lower surface of the filter were fixed and stained with the Diff‐Quick kit. The mobility was scored by counting the number of migrating cells from three randomly selected fields.

### Cytokine Array

The human cytokine array (Proteome Profiler Array Human Cytokine ARY005B; R&D Systems, Minneapolis, MN, USA) was performed according to the manufacturer's instructions. The cytokine levels from cytokine array were visualized on an X‐ray film and quantified by densitometry using the Image J software. The cytokine profiling antibody array (SCK100; Full Moon Biosystems, Sunnyvale, CA, USA) was performed according to the manufacturer's instructions. THP‐1 macrophages were treated with CM of irradiated or non‐irradiated U87MG cells for 24 h. After CM treatment, each cell was incubated in serum‐free media for 24 h. Proteins were extracted from media to perform the cytokine profiling antibody array. The slide scanning was performed using GenePix 4100A scanner (Axon Instrument, USA). After getting the scanned image, they were grided and quantified with GenePix 7.0 Software (Axon Instrument, USA). The data about protein information was annotated using UniProt DB. Data mining and graphic visualization were performed using ExDEGA (Ebiogen Inc., Korea).

### ELISA

sICAM‐1 levels were quantified by using mouse and human ICAM‐1 ELISA Kit (R&D Systems) according to the manufacturer's protocol.

### Flow Cytometric Analysis

For the flow cytometric analysis, a total of 1 × 106 control cells and/or appropriate condition treated cells were collected with trypsin digestion, and then washed and re‐suspended with 1 × PBS. Subsequently, the cells were stained with R‐phycoerythrin (PE)‐conjugated anti‐CD44 monoclonal antibody and FITC‐conjugated anti‐CD24 antibody (Miltenyi Biotec, Inc., Bergisch Gladbach, Germany) at 4 °C for 30 min. The data were analyzed using CellQuest software (BD Biosciences). All the experiments were repeated three times.

### Irradiation

GBM cells or mouse brains were exposed to *γ*‐rays using a ^137^Cs *γ*‐ray source (Atomic Energy of Canada Limited, Mississauga, Canada) at a dose rate of 3.81 Gy min^−1^.

### Transfection

Cells were transfected with DNA expression vectors or siRNAs using a Microporator mini (Digital Bio Technology, Seoul, Korea) according to the manufacturer's instructions.

### Active MMP9 Assay

Conditioned medium from U87MG cells irradiated or not was examined to validate MMP9 activity. The active MMP9 assay (Human Active MMP‐9 Fluorokine E kit F9M00; R&D Systems, Minneapolis, MN, USA) was performed according to the manufacturer's instructions.

### IHC

Mouse or tissues from patients with GBM were fixed in formalin for the preparation of paraffin sections. Paraffin‐embedded tissue sections were deparaffinized in xylene, and 95, 90, and 70% ethanol, followed by PBS. Epitopes were unmasked with 20 mg mL^−1^ proteinase K in PBS with 0.1% Triton X‐100. Sections were stained with H&E or immunostained overnight at 4 °C with a primary antibody. After washing in PBS, biotinylated goat anti‐rabbit IgG or anti‐mouse IgG antibody was then applied to the sections for 30 min. After washing in PBS, the ABC reagent (Vector Laboratories, Burlingame, CA, USA) was applied to the sections for 30 min. The color reaction was performed with 3, 3′‐diaminobenzidine (Vector Laboratories). After counter‐staining with hematoxylin and clearing with graded ethanol series and xylene, the sections were mounted with Canada balsam. Images were captured by a DP71 digital imaging system on an IX71 microscope (Olympus, Seoul, Korea). Stained slides were scored according to the intensity of staining for each antigen (low: 1–3; intermediate: 4–6; strong: 7–10). The intensity score was used for statistical analysis.

### Immunocytochemistry

Cells were fixed with 4% paraformaldehyde and permeabilized with 0.1% Triton X‐100 in phosphate buffered saline (PBS). Following fixation, cells were incubated at 4 °C overnight with a primary antibody in PBS with 1% bovine serum albumin and 0.1% Triton X‐100. Stained proteins were visualized using Alexa Fluor 488‐ or 594‐conjugated anti‐rabbit or anti‐mouse secondary antibodies (Molecular Probes, Seoul, Korea). Nuclei were counterstained with DAPI (Sigma‐Aldrich). Stained cells were observed by using an Olympus IX71 fluorescence microscope (Olympus, Seoul, Korea).

### Statistical Analysis

All experimental data are reported as mean ± standard deviation (SD, represented by error bars), and all experiments were repeated three times. *β*‐actin was used as control for normalization of expression in qRT‐PCR. Statistical analysis was performed by using the parametric Student's *t*‐test to compare between two groups. *P*‐values which were less than 0.05 were considered significant (n.s, non‐significant; *, *p* < 0.05; **, *p* < 0.01; ***, *p* < 0.001). Sample size for each statistical analysis was indicated. GraphPad Prism software 9.0 was used for statistical analysis.

## Conflict of Interest

The authors declare no conflict of interest.

## Author Contributions

K.‐C.Y. and J.‐H.K. contributed equally to this work. K.‐C.Y., Y.J.S., and S.‐J.L. conceptualized the study, designed the experiments, and wrote the manuscript. J.‐H.K., M.‐Y.C. designed the proteomic analysis and evaluated the results and performed GSEA. K.‐C.Y. and M.‐J.K. performed the biochemical assays and the in vitro and in vivo testing experiments. J.‐H.K. and Y.Z. prepared the figures. J.H.C., J.‐K.S., and S.‐J.Y. performed the clinicopathological characterization of animal experimental tumor models. S.‐G.K. provided clinical advice.

## Supporting information

Supporting Information

## Data Availability

Research data are not shared.
