# Supplementary material for: Soluble ICAM‐1 a Pivotal Communicator between Tumors and Macrophages, Promotes Mesenchymal Shift of Glioblastoma
Source: Adv Sci (Weinh). 2021 Nov 23;9(2):2102768. doi: 10.1002/advs.202102768 (PMC8805565; doi:10.1002/advs.202102768)
Supplement: Supplementary file 1 — Supporting Information [file ADVS-9-2102768-s001.pdf]

WILEY-VCH

ADVANCED SCIENCE
Open Access

Supporting Information

for *Adv. Sci.*, DOI: 10.1002/advs.202102768

Soluble ICAM-1 a Pivotal Communicator between Tumors and Macrophages, Promotes Mesenchymal Shift of Glioblastoma

Ki-Chun Yoo[1,2,†], Jae-Hyeok Kang[1,†], Mi-Young Choi[1,†], Yongjoon Suh[1], Yi Zhao[1], Min-Jung Kim[3], Jong Hee Chang[4], Jin-Kyoung Shim[4], Seon-Jin Yoon[4], Seok-Gu Kang[4,], Su-Jae Lee[1,*]*

Supporting Information

Soluble ICAM-1 a pivotal communicator between tumors and macrophages, promotes mesenchymal shift of glioblastoma

Ki-Chun Yoo[1,2,†], Jae-Hyeok Kang[1,†], Mi-Young Choi[1,†], Yongjoon Suh[1], Yi Zhao[1], Min-Jung Kim[3], Jong Hee Chang[4], Jin-Kyoung Shim[4], Seon-Jin Yoon[4], Seok-Gu Kang[4,], Su-Jae Lee[1,]**

[1]Department of Life Science, Research Institute for Natural Sciences, Hanyang University, Seoul, Korea.
[2]Department of Lymphoma and Myeloma, Division of Cancer Medicine, Center for Cancer Immunology Research, The University of Texas MD Anderson Cancer Center, Houston, TX 77030, USA.
[3]Laboratory of Radiation Exposure & Therapeutics, National Radiation Emergency Medical Center, Korea Institute of Radiological and Medical Sciences, Seoul 01812, Korea.
[4]Department of Neurosurgery, Brain Tumor Center, Severance Hospital, Yonsei University College of Medicine, Seoul 03722, Korea.

† These authors equally contributed in this work.

***Correspondence should be addressed to :** Su-Jae Lee, Ph.D
Laboratory of Molecular Biochemistry, Department of Life Science, Hanyang University, 222 Wangsimni-ro, Seongdong-gu, Seoul 04763, Korea.
Phone: 82-2-2220-2557; Fax: 82-2-2299-0762;
E-mail: sj0420@hanyang.ac.kr

; Seok-Gu Kang, M.D, Ph.D
Department of Neurosurgery, Brain Tumor Center, Severance Hospital, Yonsei University College of Medicine, Seoul 03722, Korea
Phone: 82-2-2228-2150; Fax: 82-2-393-9979;
E-mail: seokgu9@gmail.com

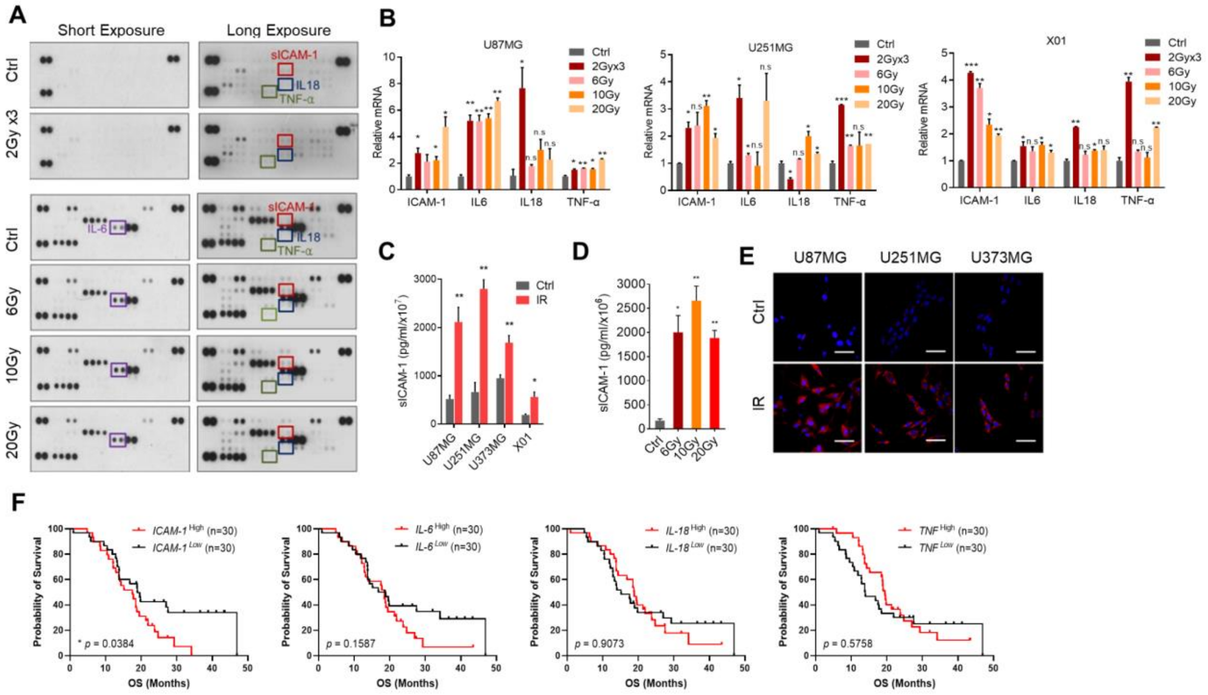

Figure S1: Radiation induces secretion and expression of ICAM-1 in GBM

(A) Cytokine array on the conditioned medium of U87MG cells exposed to radiation with either a fractionated dose, 2 Gy/d × 3 (2 Gy per day for 3 days), or single dose (6, 10, or 20 Gy).

(B) RT-qPCR of *ICAM-1*, *IL-6*, *IL-18*, and *TNF-α* in U87MG, U251MG, and X01 cells after radiation as indicated. Data are presented as mean ± SD.

(C) ELISA for sICAM-1 level in four different GBM cell lines after fractionated radiation (2Gy per day for 3 days).

(D) ELISA for sICAM-1 level in U87MG cells after various single dose of radiation.

(E) Immunocytochemical analysis of ICAM-1 in U87MG, U251MG, and U373MG GBM cells after radiation.

(F) Overall survival curves for *ICAM-1*, *IL-6*, *IL-18*, and *TNF-α* in patients with GBM based on low and high expression (GSE74187, n= 60)

Data are presented as mean ± SD. β-actin was used as control for normalization of expression. n.s, non-significant; *, $p < 0.05$ vs. control; **, $p < 0.01$ vs. control; ***, $p < 0.001$ vs. control. A two-tailed Student's t-test was used to compare data between two groups.

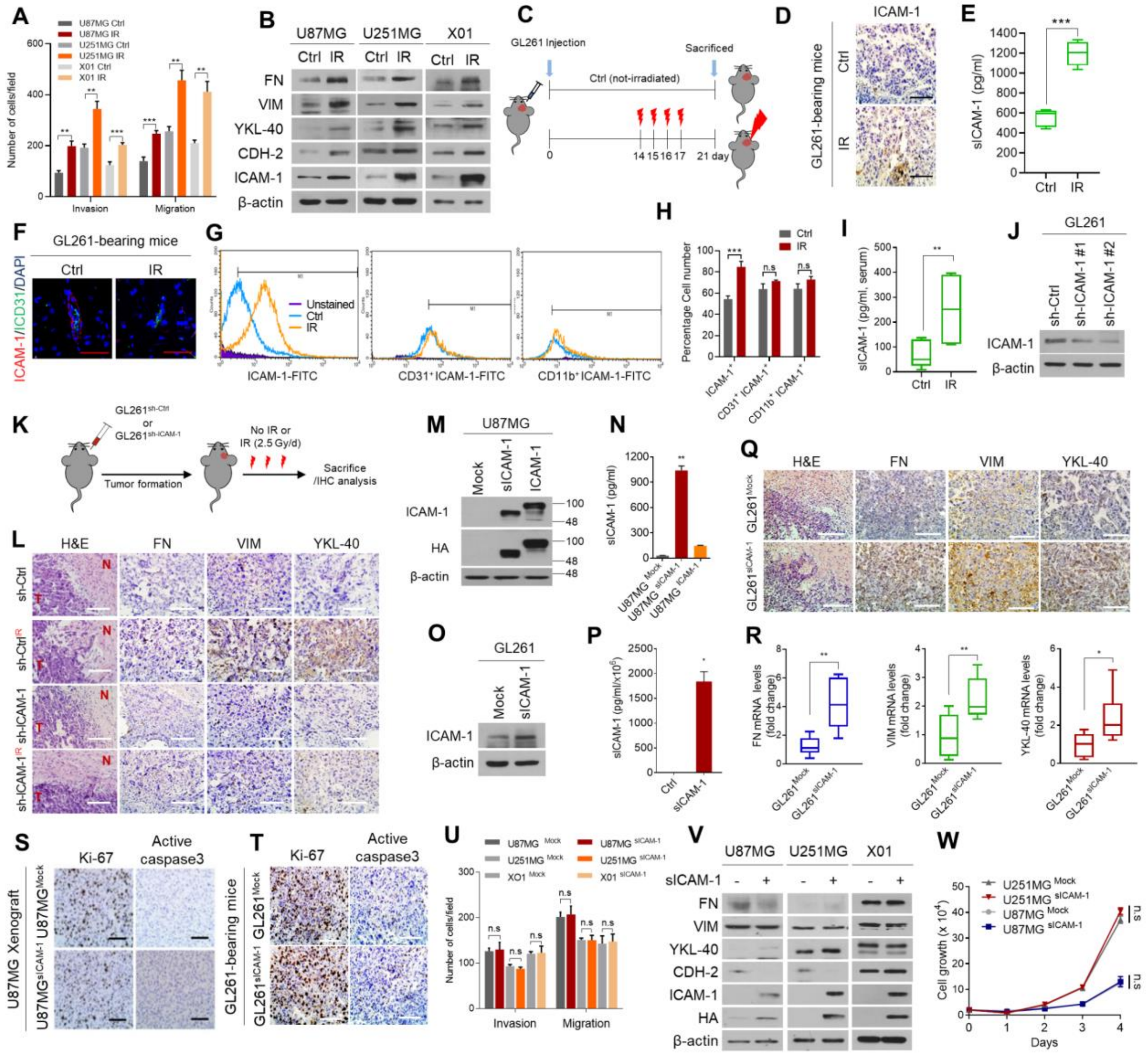

Figure S2: Radiation-induced mesenchymal shift of GBM is mediated by soluble ICAM-1

(A, B) Invasion, migration assay **(A)** and western blot analysis **(B)** of mesenchymal markers in GBM cell lines after radiation.

(C) Schematic of the animal experimental design. GL261 cells were orthotopically injected into C57BL/6 mice (n = 7 per group).

(D, E) IHC analysis of ICAM-1 **(D)** and ELISA of sICAM-1 level **(E)** in GL261 orthotopic syngeneic tumor in C57BL/6 after cranial irradiation. Scale bar, 200μm.

(F) IHC analysis of CD31 and ICAM-1 in GL261 orthotopic syngeneic tumors irradiated (2.5 Gy per day for 3 days) or not irradiated. Scale bar, 100μm.

(G, H) FACS analysis was perfomred using GL261 orthotopic syngeneic tumors to assess ICAM-1 expression in tumors, endothelial cells, and macrophages.

(I) The amount of sICAM-1 in the blood extracted from Heart in GL261-bearing syngeneic mice.

(J) Western blot analysis for the silencing efficiency of ICAM-1 in GL261 cells transfected with two different sequences of ICAM-1 shRNA.

(K) Schematic of the animal experimental design. GL261$^{sh\text{-}Ctrl}$ or GL261$^{sh\text{-}ICAM\text{-}1}$ cells were orthotopically injected into C57BL/6 mice (n = 7 per group).

(L) H&E and IHC analysis of FN, VIM, and YKL-40 in GL261$^{sh\text{-}Ctrl}$ or GL261$^{sh\text{-}ICAM\text{-}1}$ orthotopic syngeneic tumors after irradiation. Scale bar, 200μm.

(M) Expression constructs encoding sICAM-1 and western blot analysis for validation of exogenous expression of ICAM-1 and sICAM-1 in U87MG cells.

(N) ELISA of sICAM-1 in U87MGMock, U87MGsICAM-1, and U87MGICAM-1.

(O) Western blot analysis for validation of exogenous expression of sICAM-1 in GL261 cells.

(P) ELISA of sICAM-1 in GL261^{Mock} and GL261$^{sICAM\text{-}1}$.

(Q) H&E and IHC analysis of FN, VIM, and YKL-40 in GL261^{Mock} and GL261$^{sICAM\text{-}1}$ orthotopic syngeneic tumor (n = 7 per group). Scale bar, 200μm.

(R) RT-qPCR of *FN*, *VIM*, and *YKL-40* in GL261^{Mock} and GL261$^{sICAM\text{-}1}$ orthotopic syngeneic tumors.

(S) IHC analysis of Ki-67 and active caspase-3 in U87MG^{Mock} and U87MG$^{sICAM\text{-}1}$ orthotopic xenograft tumors. Scale bar, 200μm.

(T) IHC analysis of Ki-67 and active caspase-3 in GL261^{Mock} and GL261$^{sICAM\text{-}1}$ syngeneic tumors. Scale bar, 200μm.

(U) Migration and invasion assay of GBM cells transfected with mock or sICAM-1 construct.

(V) Western blot analysis of mesenchymal markers in GBM cell lines after transfection with *sICAM-1*.

(W) Quantification of cell growth of GBM cells after transfection with mock or *sICAM-1* construct.

Data are presented as mean ± SD. β-actin was used as control for normalization of expression. n.s, non-significant; *, $p < 0.05$ vs. control; **, $p < 0.01$ vs. control; ***, $p < 0.001$ vs. control. A two-tailed Student's t-test was used to compare data between two groups.

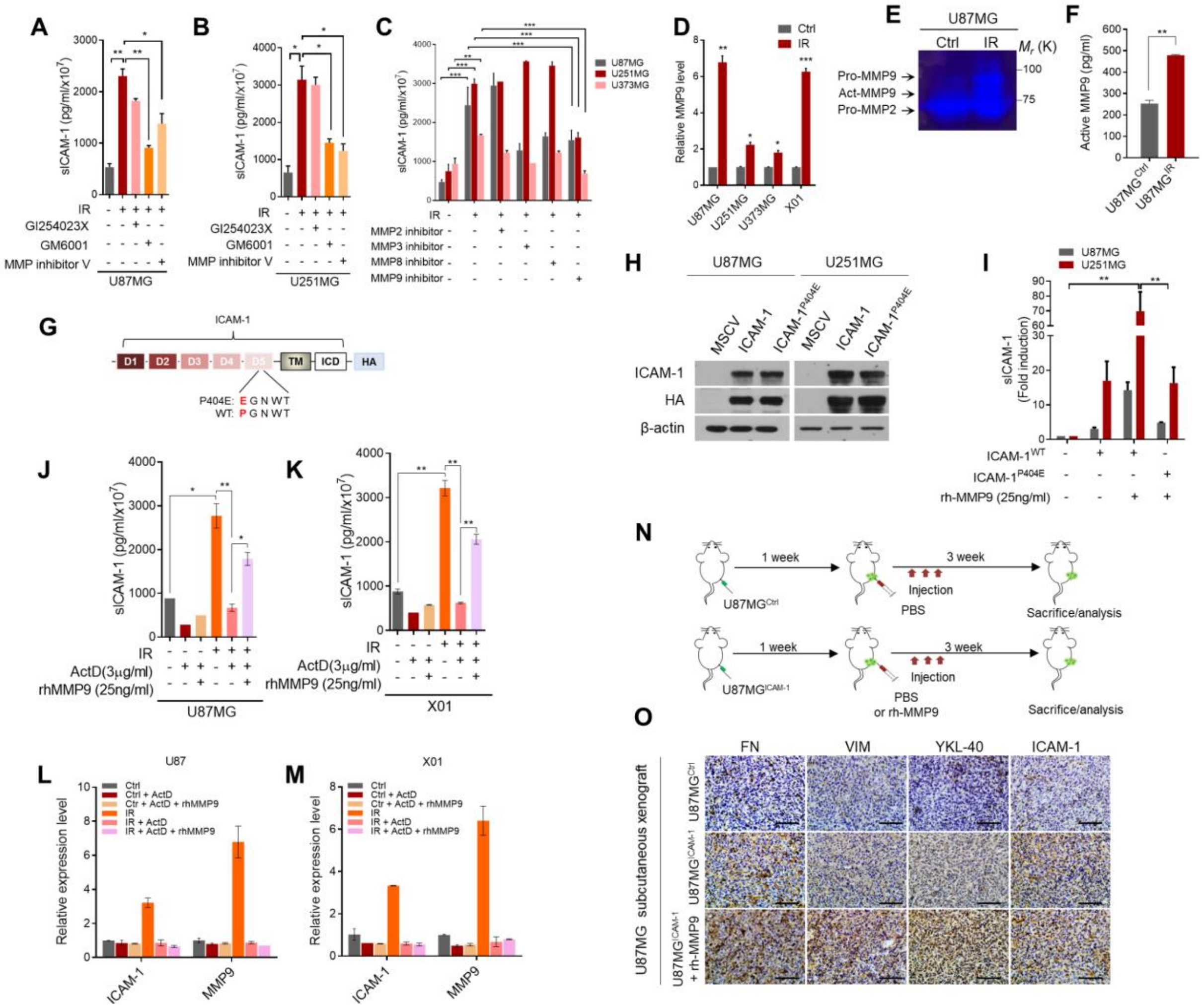

Figure S3: Radiation promotes the expression of MMP-9 that catalyzes the proteolytic cleavage of membrane-bound ICAM-1

(A, B) Quantification by ELISA of sICAM-1 in U87MG **(A)** and U251MG **(B)** cells after treatment with the selective ADAM10 inhibitor GI254023X or MMP inhibitor GM6001 prior to radiation as indicated.

(C) Quantification by ELISA of sICAM-1 level in U87MG, U251MG, or U373MG cells after treatment with inhibitors against MMP-2, -3, -8 or -9 prior to radiation.

(D) RT-qPCR analysis of *MMP-9* in U87MG, U251MG, U373MG, and GBM patient-derived X01 cells after radiation.

(E, F) Analysis of MMP9 activity by zymography **(E)** and active MMP9 assay **(F)** in U87MG cells after radiation.

(G) Schematic diagram of expression constructs encoding ICAM-1^{WT} and ICAM-1^{P404E}.

(H) Western blot analysis of ICAM-1WT or ICAM-1^{P404E} expression in U87MG and U251MG cells.

(I) Quantification by ELISA of sICAM-1 level in U87MG or U251MG cells after transfection with ICAM-1WT or ICAM-1P404E and/or following treatment with rh-MMP-9 as indicated.

(J, K) Quantification by ELISA of sICAM-1 level in U87MG **(J)** and X01 **(K)** cells after treatment with ActD and rh-MMP9 prior to radiation.

(L, M) RT-PCR analysis for *ICAM-1* and *MMP9* in U87MG **(L)** and X01 **(M)** cells after treatment with ActD and/or rh-MMP9 Prior to radiation.

(N) Schematic of the animal experimental design. U87MG^{Ctrl} or U87MG$^{ICAM\text{-}1}$ cells were subcutaneously injectred into BALB/c nude mice (n = 4 per group).

(O) IHC analysis of FN, VIM, YKL-40, and ICAM-1 in U87MG subcutaneous xenograft tumors. Scale bar, 200μm.

Data are presented as mean ± SD. β-actin was used as control for normalization of expression. n.s, non-significant; *, $p < 0.05$ vs. control; **, $p < 0.01$ vs. control; ***, $p < 0.001$ vs. control. A two-tailed Student's t-test was used to compare data between two groups.

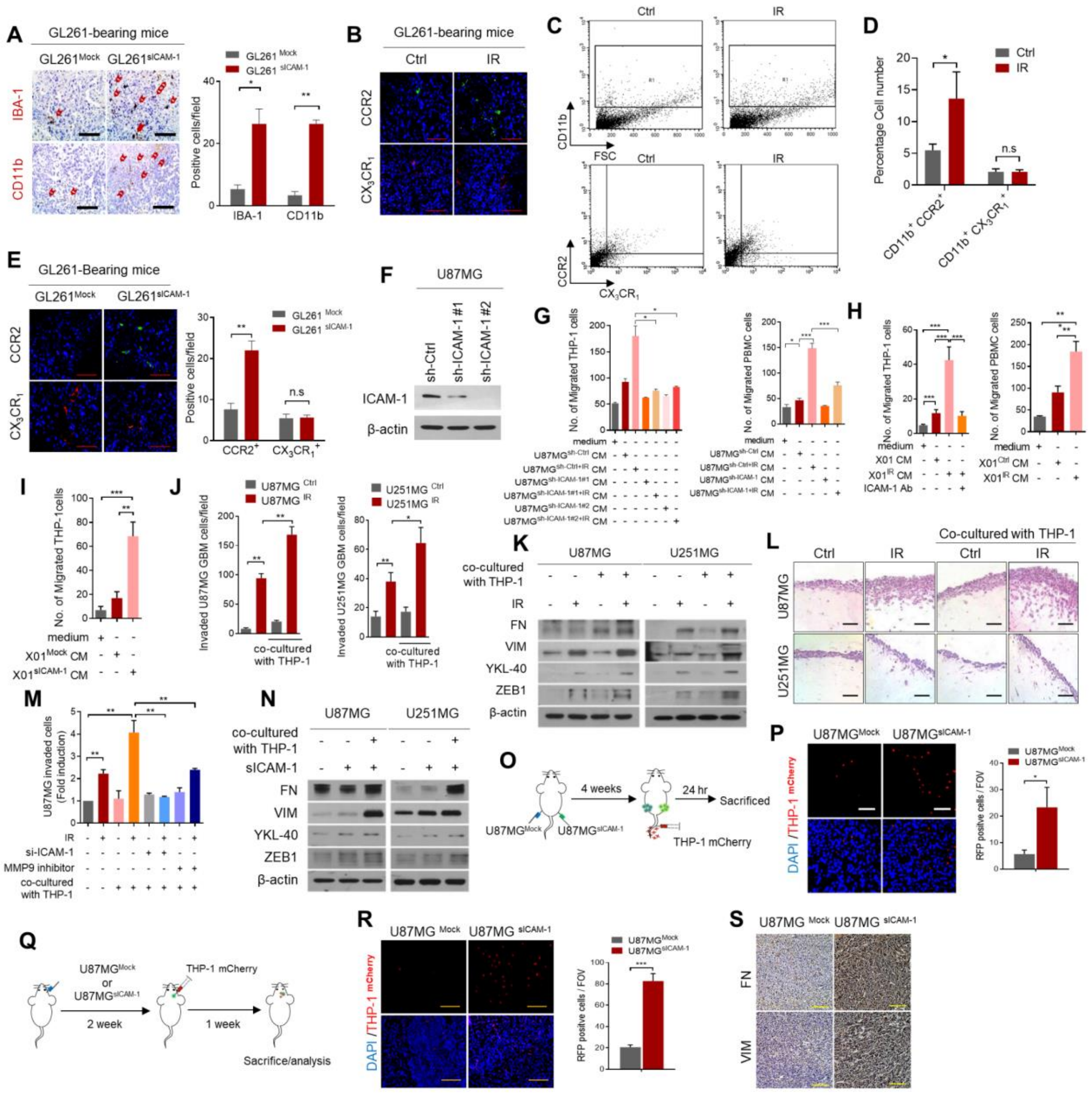

Figure S4: sICAM-1 chemoattracts inflammatory macrophages into GBM

(A) Representative photos and graphical quantification of IHC analysis for IBA-1 and CD11b in GL261^{Mock} or GL261$^{sICAM-1}$ orthotopic syngeneic tumors (n = 7 per group). Scale bar, 200µm.

(B) IHC analysis for CCR2 and CX_3CR_1 in GL261 orthotopic syngeneic tumors irradiated (2.5 Gy per day for 3 days) or not irradiated (n = 7 per group). Scale bar, 200µm.

(C, D) FACS analysis was performed using GL261 ortotopic syngeneic tumors to assess the infiltrated monocytes, macrophages ($CD11b^+CCR2^+$) versus microglia cells ($CD11b^+CX_3CR_1^+$).

(E) IHC analysis of CCR2 and CX_3CR_1 in GL261^{Mock} or GL261$^{sICAM-1}$ orthotopic syngeneic tumors and graphical quantification of CCR2- or CX_3CR_1-expressing cells. Scale bar, 200µm.

(F) Western blot analysis for the silencing efficiency of ICAM-1 in U87MG cells transfected with two different sequences of ICAM-1 shRNA.

(G) Quantification of mobility of THP-1 macrophages and PBMCs in CM of U87MG cells transfected with ICAM-1 shRNA (sh-ICAM-1) or control shRNA (sh-Ctrl) prior to irradiation.

(H) Quantification of mobility of THP-1 macrophages and PBMCs in the conditioned medium (CM) of X01 cells irradiated or non-irradiated, and following treatment with ICAM-1 antibody.

(I) Quantification of mobility of THP-1 macrophages in CM of X01 cells transfected with sICAM-1 or mock construct.

(J) Invasion assay of U87MG and U251MG cells in coculture with or without THP-1 macrophages.

(K) Western blot analysis for FN, VIM, YKL-40, and ZEB1 in GBM cells irradiated or non-irradiated prior to coculture with THP-1 macrophages.

(L) Infiltration of U87MG or U251MG GBM cells irradiated or non-irradiated prior to coculture with THP-1 macrophages in a collagen-based matrix. Scale bar, 200μm.

(M) Invasion assay of U87MG cells treated with ICAM-1 siRNA or MMP-9 inhibitor prior to irradiation and following coculture with or without THP-1 macrophages as indicated.

(N) Western blot analysis of FN, VIM, YKL-40, and ZEB1 in U87MG and U251MG cells transfected with sICAM-1 prior to coculture with or without THP-1 macrophages.

(O) Schematic of the animal experiment design for preferential recruitment of THP-1 macrophages to $U87MG^{Mock}$ or $U87MG^{sICAM\text{-}1}$ subcutaneous xenograft tumors in BALB/c nude mice (n = 3 per group).

(P) IHC analysis of mCherry-labeled THP-1 macrophages in $U87MG^{Mock}$ or $U87MG^{sICAM\text{-}1}$ xenograft tumors. Scale bar, 100μm.

(Q) Schematic of the animal experiment design for preferential recruitment of THP-1 macrophages to $U87MG^{Mock}$ or $U87MG^{sICAM\text{-}1}$ orthotopic xenograft tumors in BALB/c nude mice (n = 3 per group).

(R, S) IHC analysis of the recruitment of THP-1 macrophages to $U87MG^{Mock}$ or $U87MG^{sICAM\text{-}1}$ orthotopic xenograft tumors **(R)** and expression of FN and VIM **(S)**. Scale bar, 200μm.

Data are presented as mean ± SD. β-actin was used as control for normalization of expression. n.s, non-significant; *, $p < 0.05$ vs. control; **, $p < 0.01$ vs. control; ***, $p < 0.001$ vs. control. A two-tailed Student's t-test was used to compare data between two groups.

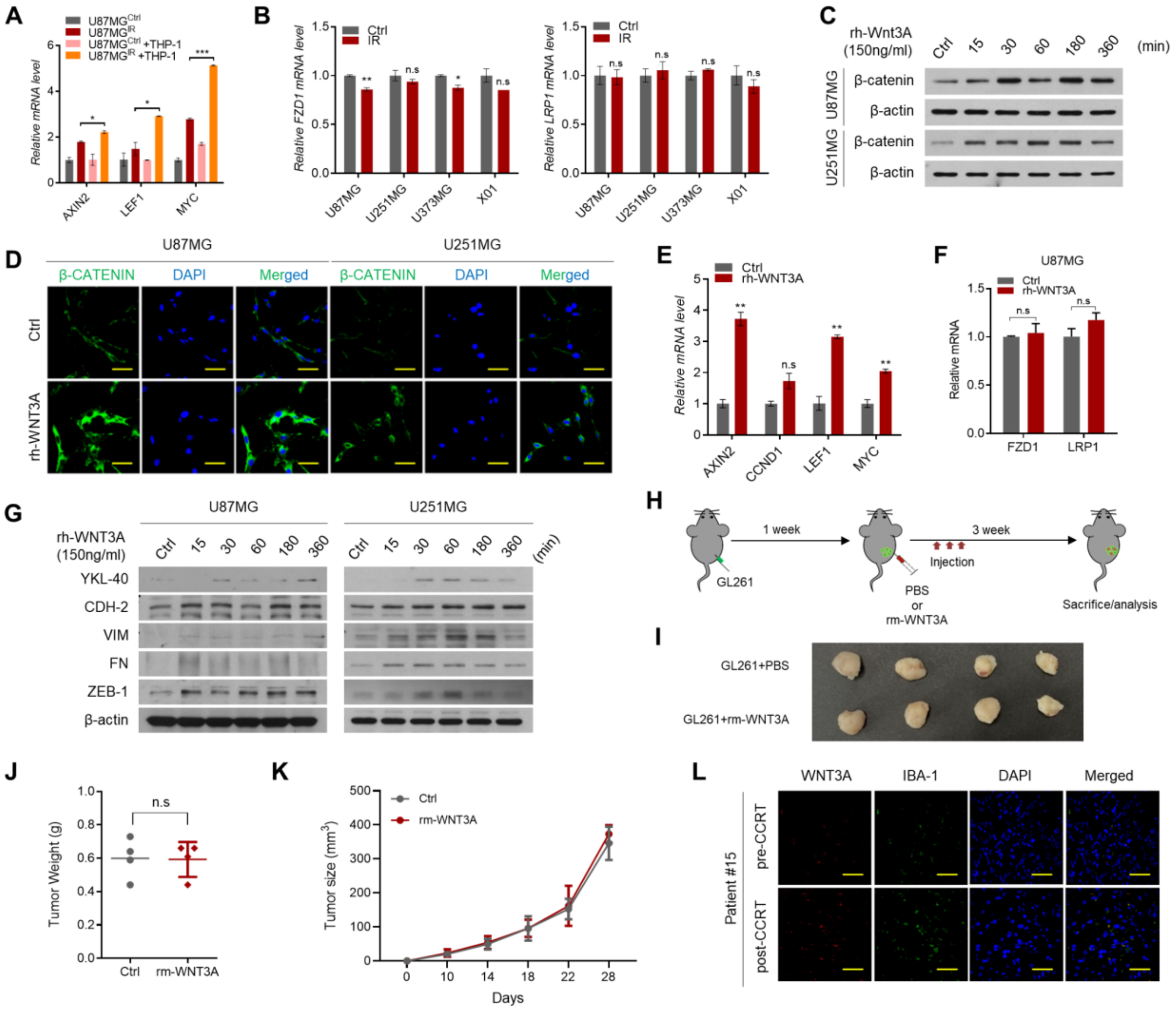

Figure S5: Secretion of WNT3A is increased in THP-1 macrophages by radiation-induced sICAM-1 and promotes mesenchymal shift of GBM cells

(A, B) RT-qPCR of target genes of β-catenin **(A)** and receptors of WNT3A **(B)** in GBM cells irradiated or non-irradiated prior to coculture with THP-1 macrophages.

(C, D) Western blot **(C)** and ICC **(D)** analysis of β-catenin in GBM cells after treatment with rh-WNT3A as indicated. Scale bar, 100μm.

(E, F) RT-PCR of target genes of β-catenin **(E)** and receptors of WNT3A **(F)** in U87MG cells after treatment with rh-WNT3A.

(G) Western blot analysis of YKL-40, CDH-2, VIM, FN, and ZEB1 in GBM cells after treatment with rh-WNT3A.

(H) Schematic of the animal experimental design. GL261 cells were subcutaneously injected into right flank of C57BL/6 mice (n = 4 per group).

(I-K) Tumor images **(I)**, weight **(J)** and growth **(K)** in subcutaneous xenograft tumors.

(L) IHC analysis of WNT3A and IBA-1 in a pair of specimens of a patient with GBM (#15) pre-CCRT and post-CCRT. Scale bar, 200μm.

Data are presented as mean ± SD. β-actin was used as control for normalization of expression. n.s, non-significant; *, $p < 0.05$ vs. control; **, $p < 0.01$ vs. control; ***, $p < 0.001$ vs. control. A two-tailed Student's t-test was used to compare data between two groups.

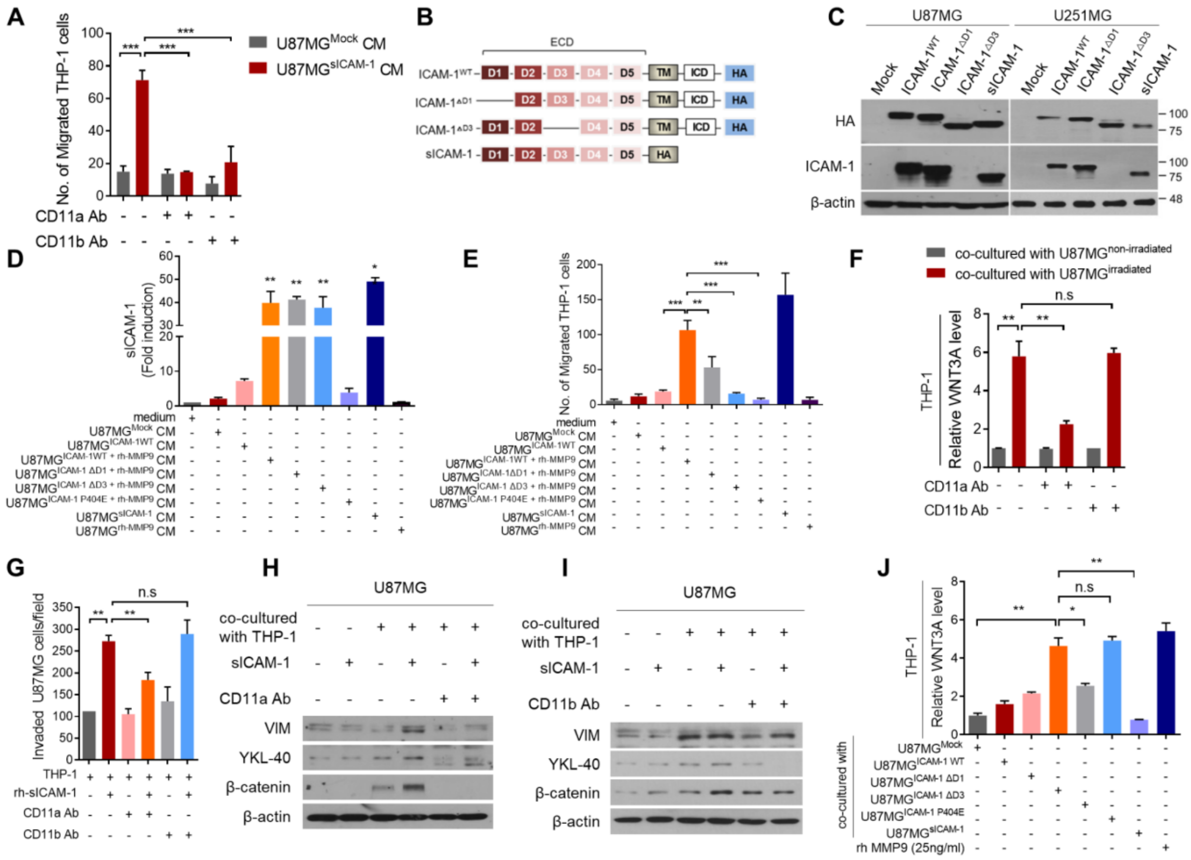

Figure S6: Extracellular domain-1 of sICAM-1 is necessary for both recruitment and WNT3A induction of macrophages, but extracellular domain-3 is only necessary for macrophage recruitment.

(A) Quantification of mobility of THP-1 macrophages in the conditioned medium (CM) of U87MG cells transfected with sICAM-1 or mock vector and following treatment with neutralizing antibody against CD11a or CD11b.

(B) Schematic diagram of expression constructs encoding ICAM-1 with or without deletion of extracellular domain-1 (*ΔD*1), -3 (*ΔD*3), or transmembrane/intracellular domain (TM/ICD; sICAM-1).

(C) Western blot analysis of expression constructs of ICAM-1 with or without deletion of extracellular domain-1(*ΔD1*), domain-3 (*ΔD3*), or transmembrane/intracellular domains (TM/ICD; sICAM-1) in U87MG and U251MG GBM cells.

(D) ELISA of sICAM-1 in conditioned medium (CM) of U87MG GBM cells transfected with each construct of ICAM-1 and following treatment with rh-MMP-9.

(E) Quantification of mobility of THP-1 macrophages in conditioned medium (CM) of U87MG cells transfected with each deletion construct of sICAM-1 and following treatment with rh-MMP-9.

(F) RT-qPCR analysis of *WNT3A* expression in THP-1 macrophages cocultured with U87MG cells irradiated or not irradiated, and following treatment with a neutralizing antibody against CD11a or CD11b.

(G) Invasion of U87MG cells cocultured with THP-1 macrophages in combination treatments with rh-sICAM-1 and/or a neutralizing antibody against CD11a or CD11b as indicated.

(H-I) Western blot analysis of VIM, YKL-40, and β-catenin in U87MG cells after coculture with THP-1 macrophages in combination treatment with sICAM-1 and/or a neutralizing antibody against CD11a **(H)** or CD11b **(I)**.

(J) RT-qPCR analysis of *WNT3A* expression in THP-1 macrophages cocultured with U87MG cells transfected with either $ICAM\text{-}1^{\mathrm{WT}}$, $ICAM\text{-}1^{\Delta D1}$, $ICAM\text{-}1^{\Delta D3}$, $ICAM\text{-}1^{\mathrm{P404E}}$, or *sICAM-1* prior to treatment with rh-MMP-9.

Data are presented as mean ± SD. β-actin was used as control for normalization of expression. n.s, non-significant; *, $p < 0.05$ vs. control; **, $p < 0.01$ vs. control; ***, $p < 0.001$ vs. control. A two-tailed Student's t-test was used to compare data between two groups.

Table S1: Cytokine profiling antibody array

Filter: 310			Fold change		Normalized data (log2)		
ID	Gene symbol	Antibody name	MΦ+U87CM /MΦ	MΦ+U87 IR CM /MΦ	MΦ	MΦ +U87CM	MΦ +U87 IR CM
1	TNFRSF9	4-1BB Receptor	0.900	0.921	8.087	7.934	7.968
2	ADIPOQ	Adiponectin	0.892	1.042	8.226	8.060	8.285
3	PROK1	EG-VEGF	0.928	0.945	8.237	8.129	8.156
4	COL18A1	Endostatin	0.883	0.958	8.077	7.898	8.015
5	CCL11	Eotaxin	1.008	1.127	7.892	7.904	8.064
6	FGF1	FGF-acidic	1.022	1.106	7.825	7.856	7.971
7	FGF2	FGF-basic	1.109	1.106	7.881	8.030	8.026
8	FLT3LG	Flt3-Ligand	1.073	1.046	7.773	7.874	7.838
9	CSF3	G-CSF	1.056	1.565	7.755	7.833	8.401
10	CSF2	GM-CSF	1.056	1.088	7.933	8.011	8.054
11	IGF1	IGF-I	1.055	1.010	7.933	8.010	7.947
12	IGF2	IGF-II	1.015	0.964	7.952	7.974	7.899
13	IL1R1	IL-1RA	1.151	1.060	9.250	9.453	9.334
14	IL1A	IL-1alpha	0.849	0.866	8.118	7.881	7.910
15	IL6	IL-6	0.963	0.958	7.942	7.888	7.879
16	IL7	IL-7	0.996	0.998	8.071	8.065	8.068
17	CSF1	M-CSF	0.953	1.033	7.961	7.891	8.008
18	PDGFA	PDGF-AA	0.891	1.017	7.963	7.797	7.987
19	PDGFB	PDGF-BB	0.878	0.959	7.943	7.755	7.883
20	CCL5	RANTES	0.923	1.150	8.323	8.208	8.524
21	KITLG	SCF	0.883	1.003	8.091	7.912	8.096
22	FASLG	sFas Ligand/Apo1L	0.833	1.356	8.814	8.551	9.254
23	IL2RA	sIL-2R alpha	0.951	1.080	8.156	8.085	8.268
24	TNFRSF11A	sRANK Receptor	0.953	1.040	7.857	7.788	7.913
25	TNFSF11	sRANKL	0.991	0.996	7.848	7.835	7.841
26	TNFRSF1A	sTNF-receptor	1.029	1.139	7.959	8.000	8.146
27	TNFRSF1B	sTNF-receptor II	1.065	1.079	8.022	8.112	8.131
28	TNF	TNF-alpha	1.053	1.163	7.983	8.057	8.201
29	LTA	TNF-beta	1.009	1.008	7.855	7.868	7.867
30	VCAM1	VCAM-1	1.019	0.985	8.391	8.418	8.369
31	EGFR	EGFR	0.895	0.910	8.254	8.094	8.118
32	IL4	IL-4	1.016	1.163	8.311	8.333	8.529
33	CD40LG	sCD40 Ligand	0.933	1.028	7.990	7.890	8.030
34	VEGFA	VEGF	0.856	0.909	8.091	7.866	7.954
35	IL2	IL-2	0.916	0.877	7.950	7.824	7.761

36	CXCL8	IL-8	1.053	0.962	7.834	7.909	7.779
37	IL10	IL-10	1.151	1.057	7.720	7.923	7.801
38	TNFSF9	4-1BBL	1.354	1.314	7.462	7.899	7.856
39	ADIPOQ	Adipolean Variant	1.242	1.295	7.581	7.894	7.954
40	TNFSF18	AITRL	1.664	1.776	7.296	8.030	8.124
41		ApoE3	1.768	2.387	7.257	8.079	8.512
42	TNFSF13	APRIL	1.494	1.509	7.254	7.833	7.848
43	ARTN	Artemin	1.715	1.693	7.342	8.120	8.102
44	TNFSF13B	BAFF	1.605	1.578	7.350	8.033	8.008
45	CXCL13	BCA-1	1.424	1.404	7.429	7.939	7.919
46	DEFB1	BD-1	1.364	1.362	7.483	7.932	7.930
47	DEFB4A	BD-2	1.317	1.311	7.517	7.913	7.907
48	DEFB103B	BD-3	1.268	1.346	7.554	7.896	7.982
49	DEFB104A	BD-4	1.264	1.241	7.531	7.869	7.842
50	BDNF	BDNF	1.085	1.092	7.716	7.835	7.844
51		Betacellulin	1.225	1.247	7.601	7.894	7.919
52	BMP2	BMP-2	1.250	1.240	7.649	7.971	7.960
53	BMP4	BMP-4	1.372	1.614	7.662	8.119	8.353
54	BMP7	BMP-7/OP-1	1.275	1.351	7.654	8.004	8.088
55	CXCL14	BRAK	1.166	1.464	7.827	8.048	8.377
56	CTF1	Cardiotrophin-1	0.949	0.965	8.033	7.958	7.982
57	CNTF	CNTF	1.198	1.209	7.714	7.976	7.988
58	CCL27	CTACK	1.289	1.287	7.586	7.952	7.949
59	CTGF	CTGF	1.448	1.473	7.439	7.973	7.998
60		CTGFL/WISP-2	2.507	2.797	7.394	8.720	8.878
61	CXCL16	CXCL16	1.635	1.546	7.360	8.069	7.989
62	AIMP1	EMAP-II	0.911	0.933	8.363	8.228	8.262
63	CXCL5	ENA-78	1.085	1.044	8.007	8.125	8.068
64	CCL24	Eotaxin-2	0.963	1.057	8.101	8.046	8.180
65	CCL26	Eotaxin-3	0.989	1.182	8.207	8.191	8.448
66	CCL21	Exodus-2	1.100	1.144	7.940	8.077	8.134
67	FGF10	FGF-10	1.094	1.693	7.983	8.113	8.743
68	FGF16	FGF-16	1.104	1.151	7.904	8.047	8.107
69	FGF17	FGF-17	1.156	1.173	7.784	7.994	8.014
70	FGF4	FGF-4	1.228	1.124	8.112	8.408	8.281
71	FGF5	FGF-5	1.162	1.193	8.180	8.397	8.435
72	FST	Follistatin	1.240	1.273	7.802	8.112	8.150
73	CX3CL1	Fractalkine	1.248	1.853	7.621	7.941	8.511
74	ADIPOQ	gAcrp30/Adipolean	1.335	1.237	8.294	8.711	8.602

75	LGALS1	Galectin-1	1.362	1.328	8.135	8.581	8.544
76	LGALS3	Galectin-3	1.280	1.287	7.808	8.163	8.172
77	CXCL6	GCP-2	1.378	1.276	7.710	8.173	8.062
78	GDF3	GDF-3	1.231	1.240	7.825	8.125	8.135
79	GDNF	GDNF	1.195	1.239	7.692	7.948	8.001
80	CXCL1	GRO/MGSA	1.167	1.286	7.747	7.970	8.109
81	CXCL2	GRO-beta	1.155	1.131	7.836	8.044	8.014
82	CXCL3	GRO-gamma	1.866	1.175	7.798	8.698	8.031
83	CCL14	HCC-1	1.294	1.142	7.755	8.126	7.946
84	NRG1	Heregulin-beta1	1.113	1.095	7.831	7.985	7.962
85	CCL1	I-309	1.095	1.019	7.942	8.073	7.969
86	IFNB1	IFN-beta	0.971	0.915	8.090	8.047	7.961
87	ifnl2	IFN-lambda2	0.946	0.828	8.195	8.114	7.923
88	IGFBP1	IGF-BP1	0.968	0.949	8.429	8.383	8.354
89	IGFBP3	IGF-BP3	0.910	0.813	8.285	8.148	7.986
90	IGFBP5	IGF-BP5	0.887	0.921	8.312	8.139	8.193
91	IGFBP7	IGF-BP7	0.999	1.283	8.299	8.297	8.659
92	IL11	IL-11	1.052	1.300	8.123	8.196	8.501
93	IL12A	IL-12	1.308	1.151	8.231	8.619	8.435
94	IL13	IL-13	0.758	0.653	8.635	8.235	8.021
95	IL15	IL-15	1.110	0.965	8.424	8.575	8.372
96	IL16	IL-16	1.159	0.971	8.143	8.356	8.100
97	IL17A	IL-17 (IL-17A)	1.085	1.159	8.048	8.166	8.261
98	IL17B	IL-17B	1.071	1.132	8.133	8.232	8.313
99	IL17D	IL-17D	1.098	1.057	8.076	8.211	8.156
100	IL25	IL-17E	1.132	1.052	8.236	8.415	8.308
101	IL17F	IL-17F	1.198	1.184	7.782	8.043	8.026
102	IL19	IL-19	1.277	1.233	7.888	8.241	8.190
103	IL20	IL-20	1.196	1.207	7.763	8.021	8.034
104	IL21	IL-21	1.137	1.548	7.796	7.981	8.427
105	IL22	IL-22	1.115	1.358	7.933	8.090	8.375
106	IL3	IL-3	1.229	1.227	7.692	7.989	7.987
107	IL31	IL-31	1.204	1.225	7.714	7.982	8.008
108	IL33	IL-33	1.181	1.223	7.737	7.977	8.028
109	IL5	IL-5	1.147	1.181	7.790	7.988	8.030
110	IL9	IL-9	1.164	1.263	7.718	7.938	8.056
111	CXCL10	IP-10	1.192	1.252	8.395	8.649	8.720
112	CXCL11	I-TAC	1.278	1.204	7.679	8.033	7.947
113	FGF7	KGF	1.092	1.140	7.819	7.946	8.009

114	CCL3L1	LD78beta	0.750	1.334	11.651	11.237	12.066
115	CCL16	LEC	1.252	1.126	7.751	8.075	7.922
116	LEP	Leptin	1.235	1.161	8.014	8.318	8.229
117	TNFSF14	LIGHT	1.125	1.150	7.924	8.094	8.126
118	XCL1	Lymphotactin	1.022	0.966	8.102	8.133	8.052
119	SERPINB5	Maspin	0.897	1.061	8.343	8.186	8.428
120	CCL2	MCP-1/MCAF	0.698	1.127	8.903	8.384	9.076
121	CCL8	MCP-2	0.648	0.621	8.777	8.150	8.090
122	CCL7	MCP-3	1.010	1.097	8.583	8.598	8.718
123	CCL13	MCP-4	1.303	1.218	8.359	8.741	8.643
124	CCL22	MDC	1.353	1.191	7.767	8.203	8.019
125	CCL28	MEC	1.380	1.185	7.877	8.342	8.122
126	MIA	MIA	1.033	0.930	8.201	8.248	8.096
127	mia2	MIA-2	1.041	0.933	8.328	8.386	8.229
128	MDK	Midkine	1.006	0.923	8.324	8.333	8.209
129	CXCL9	MIG	0.962	0.930	8.208	8.152	8.104
130	CCL3	MIP-1alpha	0.976	1.152	8.356	8.321	8.561
131	CCL4	MIP-1beta	0.899	1.252	9.143	8.991	9.467
132	CCL23	MIP-3	1.100	0.998	8.002	8.140	7.998
133	CCL20	MIP-3alpha	1.213	1.215	7.844	8.122	8.125
134	CCL19	MIP-3beta	1.144	1.128	7.817	8.011	7.991
135	CCL18	MIP-4	1.197	1.211	7.788	8.047	8.065
136	CCL15	MIP-5	1.205	1.519	7.714	7.984	8.317
137	NANOG	Nanog	1.184	1.204	7.743	7.986	8.010
138	PPBP	NAP-2	1.104	1.140	7.771	7.913	7.960
139	SERPINI1	Neuroserpin	1.275	1.542	8.283	8.634	8.908
140	NRTN	Neurturin	1.234	1.296	7.802	8.105	8.176
141	CLCF1	NNT-1/BCSF-3	1.148	1.222	7.817	8.017	8.107
142	NOV	NOV	1.203	1.201	7.784	8.051	8.048
143	DEFA1	NP-1	1.248	1.207	7.700	8.019	7.971
144	NTF3	NT-3	1.194	1.431	8.386	8.642	8.903
145	NTF4	NT-4	1.161	1.178	7.866	8.081	8.102
146	OSM	Oncostatin M	1.169	1.123	7.800	8.025	7.967
147	TNFRSF11B	OPG	1.114	1.145	8.063	8.219	8.258
148	SERPINE1	PAI-1	1.078	1.602	8.519	8.627	9.199
149	PSPN	Persephin	0.835	0.832	8.385	8.124	8.120
150	PF4	PF-4	0.885	0.982	8.622	8.446	8.595
151	PGF	PlGF	0.786	0.994	8.486	8.139	8.477
152	PTHLH	PTHrP	0.617	0.639	8.885	8.190	8.240

153		RELM beta	1.035	1.015	8.177	8.228	8.198
154	RETN	Resistin	1.106	1.504	8.390	8.535	8.979
155	CD22	sCD22	1.275	1.136	8.107	8.458	8.291
156	CLEC11A	SCGF-beta	1.549	1.255	7.823	8.455	8.151
157	CXCL12	SDF-1alpha	1.351	1.130	8.084	8.517	8.259
158	CXCL12	SDF-1beta	0.971	0.879	8.411	8.368	8.225
159	DLL4	sDLL-4	1.020	0.917	8.267	8.296	8.141
160	TNFRSF10B	sTRAIL Receptor-2	0.990	0.918	8.257	8.242	8.134
161		sTRAIL/APO2L	0.963	0.907	8.346	8.292	8.206
162	TNFRSF13B	TACI	1.030	0.962	8.214	8.257	8.159
163	CCL17	TARC	1.039	0.936	8.090	8.145	7.994
164	CCL25	TECK	1.021	1.214	8.120	8.149	8.399
165	TFF2	TFF-2	1.338	1.098	8.378	8.799	8.513
166	TGFA	TGF-alpha	1.070	1.090	7.961	8.058	8.085
167	TGFB1	TGF-beta1	1.017	1.353	8.637	8.662	9.073
168	TIMP1	TIMP-1	1.531	1.336	13.764	14.379	14.183
169	TNFSF15	TL-1A	1.232	1.212	8.138	8.439	8.416
170	THPO	TPO	1.076	1.079	7.884	7.991	7.994
171	TSLP	TSLP	1.098	1.064	7.894	8.028	7.983
172	TNFSF12	TWEAK	1.167	1.106	7.765	7.987	7.910
173	RLN3	Vaspin	1.189	1.141	7.840	8.090	8.031
174	NAMPT	Visfatin	1.179	1.301	8.028	8.266	8.408
175	WNT1	WNT-1	1.119	1.222	7.829	7.991	8.118
176	WNT3A	WNT-3a	1.163	1.361	7.894	8.111	8.338
177	NGF	NGF beta	1.185	1.149	7.940	8.185	8.140
178	APC	APC	1.119	1.482	8.640	8.802	9.207
179	CTNNA1	Catenin-alpha1	1.731	1.157	9.554	10.345	9.764
180	JUP	Catenin-gamma	0.708	1.287	9.729	9.231	10.093
181	CDH1	E-cadherin	0.630	0.893	9.700	9.034	9.538
182	CDH1	Cadherin-pan	0.639	0.735	8.793	8.147	8.349
183	ERBB3	HER3	0.772	1.075	9.425	9.051	9.529
184	FGFR1OP	FGFR1 Oncogene Partner	0.884	0.978	9.258	9.079	9.225
185	FGFR2	FGFR2	0.730	1.052	9.002	8.548	9.075
186	FGFR3	FGFR3	0.678	1.067	9.071	8.511	9.164
187	FLI1	FLI1	0.658	1.008	9.037	8.435	9.049
188	GZMB	Granzyme B	0.750	1.263	8.856	8.441	9.193
189	HDAC1	HDAC1	0.861	1.442	8.703	8.487	9.231
190	HDAC10	HDAC10	0.784	0.727	8.735	8.384	8.276
191	HDAC3	HDAC3	0.927	0.746	8.825	8.715	8.401

192	HDAC5	HDAC5	0.804	0.720	9.035	8.720	8.562
193	HDAC6	HDAC6	0.828	0.784	8.874	8.601	8.523
194	HDAC7	HDAC7	0.724	0.818	9.003	8.538	8.713
195	HDAC9	HDAC9	0.980	1.014	8.941	8.912	8.961
196	NRG1	Heregulin	1.050	1.044	9.222	9.292	9.284
197	ITGB5	Integrin beta5	1.111	1.085	8.725	8.876	8.843
198	MMP1	MMP-1	1.137	1.441	8.638	8.824	9.165
199	MMP10	MMP-10	1.521	1.172	10.123	10.728	10.352
200	MMP11	MMP-11	1.067	1.159	9.072	9.166	9.285
201	MMP13	MMP-13	1.156	1.362	8.369	8.578	8.815
202	MMP14	MMP-14	0.861	1.226	8.485	8.269	8.779
203	MMP15	MMP-15	0.769	1.214	8.596	8.217	8.875
204	MMP16	MMP-16	0.886	1.069	8.375	8.200	8.470
205	MMP19	MMP-19	0.889	1.287	8.651	8.482	9.015
206	MMP2	MMP-2	1.930	1.248	10.867	11.815	11.187
207	MMP23B	MMP-23	0.895	1.254	8.352	8.192	8.678
208	MMP3	MMP-3	0.944	1.522	8.360	8.278	8.966
209	MMP7	MMP-7	1.023	1.489	8.459	8.492	9.033
210	MMP8	MMP-8	0.862	1.307	8.786	8.571	9.173
211	MMP9	MMP-9	0.945	1.347	8.286	8.204	8.717
212	SPP1	Osteopontin	0.961	1.116	8.523	8.466	8.681
213	PDGFB	PDGFB	0.511	0.791	9.367	8.400	9.030
214	PDGFRA	PDGFR alpha	0.595	0.963	9.122	8.373	9.067
215	S100A1	S100 A1	0.565	0.857	9.273	8.450	9.051
216	TGFBR2	TGF beta Receptor II	0.749	1.083	9.106	8.690	9.221
217	TGFBR3	TGF beta Receptor III	0.783	0.999	9.205	8.851	9.203
218	TGFB2	TGF beta2	0.869	1.023	8.979	8.776	9.012
219	TGFB3	TGF beta3	0.719	1.101	8.856	8.381	8.995
220	TIMP2	TIMP2	0.744	1.020	8.771	8.343	8.799
221	TIMP3	TIMP3	0.915	1.085	8.555	8.427	8.673
222	CD40	CD40	0.873	0.705	9.588	9.392	9.084
223	FAS	FAS	0.671	0.644	9.254	8.678	8.620
224	NCOR1	NCoR1	0.588	0.527	9.402	8.636	8.478
225	TIMP4	TIMP4	0.661	0.602	9.238	8.640	8.505
226	TRADD	TRADD	0.628	0.658	9.127	8.456	8.523
227	TYR	Tyrosinase	0.861	0.749	9.044	8.829	8.626
228	RPS27A	Ubiquitin	0.912	0.887	8.394	8.261	8.221
229	VEGFB	VEGFB	1.184	1.171	8.833	9.077	9.062
230	FER	FER	1.070	1.086	8.820	8.917	8.939

231	CTNNB1	Catenin-beta 1	1.284	1.480	7.987	8.347	8.552
232	STAT3	STAT3	1.117	1.020	8.107	8.266	8.136
233	STAT1	STAT1	1.132	1.115	8.199	8.378	8.356
234	STAT5A	STAT5A	1.020	1.328	8.572	8.600	8.981
235	ERCC6	ERCC6	1.541	1.505	8.824	9.448	9.414
236	ERBB2	HER2	1.100	1.392	8.491	8.628	8.968
237	STAT5A	STAT5A/B	1.224	1.364	8.601	8.893	9.049
238	APOF	Apolipoprotein F (APOF)	1.229	1.174	7.928	8.226	8.159
239	APOL1	Apolipoprotein L1 (APOL1)	1.070	1.108	7.968	8.065	8.116
240	APOL2	Apolipoprotein L2 (APOL2)	1.088	1.195	8.085	8.207	8.342
241	BCL10	BCL-10	0.940	0.950	8.079	7.989	8.005
242	IFNG	IFN-gamma	0.985	0.968	8.005	7.984	7.958
243	CD14	CD14	0.894	0.907	8.112	7.950	7.970
244	BLK	BLK	0.694	0.668	8.605	8.078	8.022
245	CIB1	CIB1	0.591	0.554	8.776	8.016	7.923
246	EIF4EBP1	4E-BP1	0.686	0.717	8.729	8.186	8.248
247	S100A10	S 100A10/P11	0.946	0.804	8.236	8.155	7.921
248	KIT	C-Kit	0.906	0.782	8.721	8.579	8.367
249	PTK6	PTK6	0.754	0.657	8.582	8.176	7.977
250	LYN	LYN	0.767	0.683	8.517	8.134	7.967
251	GSK3A	GSK3 alpha	0.741	0.665	8.592	8.160	8.003
252	TYRO3	TYRO3	0.692	0.662	8.742	8.211	8.146
253	IGF1R	IGF 1R	0.650	0.602	9.066	8.446	8.333
254	S100B	S 100B	0.527	0.507	9.295	8.371	8.316
255	KMT2A	MLL	0.477	0.459	9.368	8.301	8.246
256	YES1	YES 1	0.659	0.491	9.202	8.601	8.175
257	GATA3	GATA3	0.509	0.423	9.356	8.380	8.115
258	SRC	SRC	0.484	0.416	9.270	8.223	8.005
259	IGFBP2	IGF-BP2	0.902	0.721	8.455	8.306	7.983
260	ALCAM	ALCAM	0.754	0.654	8.673	8.265	8.059
261	AXL	AXL	0.793	0.702	8.504	8.170	7.994
262	MST1R	RON	0.937	0.808	8.234	8.141	7.926
263	SOX2	SOX2	0.973	1.007	8.168	8.128	8.178
264	CD44	CD44	1.045	0.949	8.040	8.104	7.964
265	MPO	Myeloperoxidase (MPO)	1.100	0.971	8.064	8.202	8.022
266	MSTN	Myostatin (GDF-8)	1.109	0.944	8.028	8.178	7.945
267	PDGFRB	PDGFR beta	1.078	0.966	7.975	8.082	7.925
268	PROZ	PROZ	1.041	0.915	8.055	8.112	7.926
269	FLT1	Flt-1	1.124	1.077	7.961	8.129	8.067

270	KDR	KDR (VEGFR2)	0.972	0.921	8.091	8.051	7.973
271	ITGA5	ITGA5	0.908	0.950	8.182	8.043	8.107
272	EGF	EGF	0.877	0.920	8.305	8.116	8.184
273	NGFR	NGFR	0.774	1.173	8.400	8.030	8.631
274	AKT2	AKT2	0.531	0.606	9.168	8.256	8.445
275	GSK3B	GSK3 beta	0.811	0.768	8.418	8.116	8.038
276	ar	Androgen receptor	0.721	0.831	8.706	8.233	8.439
277	EGR1	EGR1	0.635	0.607	8.870	8.215	8.149
278	ICAM1	ICAM-1	0.830	0.698	8.595	8.326	8.075
279	GATA1	GATA1	0.831	0.708	8.558	8.291	8.060
280	EPCAM	EPCAM	0.672	0.532	8.919	8.345	8.009
281	PTK2	FAK	0.650	0.543	8.926	8.304	8.046
282	IL1B	IL-1 beta	0.780	0.840	8.856	8.498	8.605
283	AFP	AFP	0.752	0.737	8.819	8.407	8.379
284	CGA	alpha hCG	0.813	1.953	9.583	9.284	10.549
285	CGB3	beta hCG	0.770	0.852	8.936	8.558	8.704
286	B2M	Beta-2-Microglobulin	0.788	1.361	12.913	12.569	13.358
287	MUC16	CA125	1.192	0.972	8.508	8.761	8.468
288	ADAM2	CA15-3	1.220	1.128	8.265	8.553	8.439
289	IL13RA1	CA19-9	1.110	0.984	8.913	9.064	8.890
290	PSG2	CEA	1.181	1.110	8.204	8.444	8.354
291	CRP	C-reactive Protein (CRP)	1.082	1.136	8.197	8.310	8.380
292	FTH1	Ferritin	1.408	1.496	8.959	9.453	9.540
293	KLK3	Free PSA	1.176	1.158	8.053	8.287	8.264
294	FSHB	FSH	1.171	1.105	8.346	8.574	8.490
295	CGA	hCG	1.144	1.484	8.051	8.245	8.620
296	GH1	HGH	1.079	1.127	8.002	8.111	8.175
297	INS	Insulin	1.212	1.233	8.063	8.341	8.365
298	LHB	LH (Human Luteinizing Hormone)	1.139	1.134	8.055	8.242	8.235
299	PRL	Prolactin	1.153	1.089	7.995	8.201	8.118
300	KLK3	PSA-ACT	1.147	1.126	7.945	8.143	8.116
301	TG	Thyroglobulin	0.946	0.968	8.630	8.550	8.583
302	KLK3	Total PSA	0.827	0.898	8.505	8.232	8.350
303	TSHB	TSH	0.805	1.066	8.368	8.055	8.460
304	HGF	Hepatocyte Growth Factor (HGF)	0.539	0.547	9.162	8.271	8.292
305	STAT6	STAT6	0.684	0.716	9.232	8.683	8.750
306	SELE	E-Selectin	0.464	0.503	9.325	8.217	8.334
307	SELL	L-Selectin	0.655	0.734	8.885	8.276	8.440
308	ANGPT1	Angiopoietin-1	0.808	0.866	8.412	8.104	8.204

309	ANGPT2	Angiopoietin-2	0.549	0.571	8.936	8.071	8.128
310	AKT1	AKT1	0.700	1.143	8.776	8.261	8.968
